# The Spectroscopic Characterization and Photophysical Properties of a Hydrated Lanthanum Ion Complex with a Triazole Ligand by Several DFT Methods

**DOI:** 10.3390/molecules30163412

**Published:** 2025-08-18

**Authors:** M. Alcolea Palafox, Lozan T. Todorov, Nataliya P. Belskaya, Javier Álvarez-Conde, Diana Díaz-García, Santiago Gómez-Ruiz, Irena P. Kostova

**Affiliations:** 1 Departamento de Química Física, Facultad de Ciencias Químicas, Universidad Complutense, 28040 Madrid, Spain; 2 Department of Chemistry, Faculty of Pharmacy, Medical University–Sofia, 2 Dunav Str., 1000 Sofia, Bulgaria; ltodorov@pharmfac.mu-sofia.bg (L.T.T.); i.kostova@pharmfac.mu-sofia.bg (I.P.K.); 3 Department of Technology for Organic Synthesis, Ural Federal University, 19 Mira Str., Yekaterinburg 620012, Russia; n.p.belskaya@urfu.ru; 4 COMET-NANO Group, Department of Biology and Geology, Physics and Inorganic Chemistry, E.S.C.E.T., Universidad Rey Juan Carlos, Calle Tulipán s/n, 28933 Madrid, Spain; javier.alvarez@urjc.es (J.Á.-C.); diana.diaz@urjc.es (D.D.-G.); santiago.gomez@urjc.es (S.G.-R.)

**Keywords:** infrared spectra, Raman spectra, triazole ligand, DFT methods, lanthanum, photophysical properties

## Abstract

The experimental IR and Raman vibrational spectra of a hydrated La(III) complex with a 1,2,3-triazole ligand were characterized by using four different Density Functional Theory (DFT) levels and two accurate scaling procedures. In the theoretical calculations, the hydration water in the experimental sample was considered under the Discrete Model (DM) with different numbers of explicit water molecules and different positions around the La(III) ion and the carboxylate groups. The predicted IR spectra at the M06-2X/Lanl2dz level appear to be the closest to the experimental ones. Based on the optimized structures, molecular properties and global chemical descriptors were also calculated, and the findings obtained are discussed in detail herein. Additionally, several photophysical properties were determined in both the free ligand and in several lanthanide complexes, and with the sample in the solid state and in DMSO solution. A blue shift in the fluorescence of the complexes was observed compared to the free ligand, as well as in the solid-state sample compared to the solution.

## 1. Introduction

Many metal complex compounds are used today as medicinal substances and therapeutic agents. There are a huge number of potential complexing agents and numerous organic molecules in the body, which can act as bioligands through coordination bonds [1,2]. Organometallic compounds with lanthanoid (Ln(III)) ions are of interest in many fields [3,4,5], especially in cancer chemotherapy [6,7] due to their large cytotoxic activity on many different tumor cells [1,2]. Ln(III) ions tend to form stable chemical bonds with organic compounds containing O, N, or F atoms, which facilitates complex formation. Although the biological properties of such complexes have been reported in previous studies [8,9,10], there is limited data on their structural and spectroscopic characterization across the whole spectrum. Consequently, the present study addresses an organometallic complex formed with La(III) ion and a 1,2,3-triazole bioligand.

Among all possible ligands, those with heterocyclic triazoles are of particular interest. They play significant roles in chemistry, medicine, and materials science, serving as heterocyclic motifs, bioisosteric replacements for amides, carboxylic acids, and other carbonyl groups. Additionally, triazole derivatives are some of the most widely used linkers in click chemistry [11,12]. Among triazole derivatives, those with a 1,2,3-triazole ring are under intense investigation [13,14].

This heterocycle can function more efficiently as linker or bioisostere, as well as a pharmacophore of various biological activities [12], such as cytotoxic [15,16,17], antimicrobial [18], anti-inflammatory [19], analgesic and antioxidant [20], antifungal [21], and antiviral [22,23] activities, especially for anti-SARS-CoV-2 [24,25,26]. The numerous effects observed are attributed to the fact that the 1,2,3-triazole ring possesses many interesting chemical characteristics [12], such as high stability in both acidic and basic media, a high dipole moment, capacity to form H-bonds with hydrogen donors, π-π interactions with aromatic rings, and coordination bonds with metal ions [27,28]. Due to the considerable importance of this 1,2,3-triazole ring, it was chosen as one of the key moieties in the synthesis of the ligand presented in this work.

**Figure 1:**
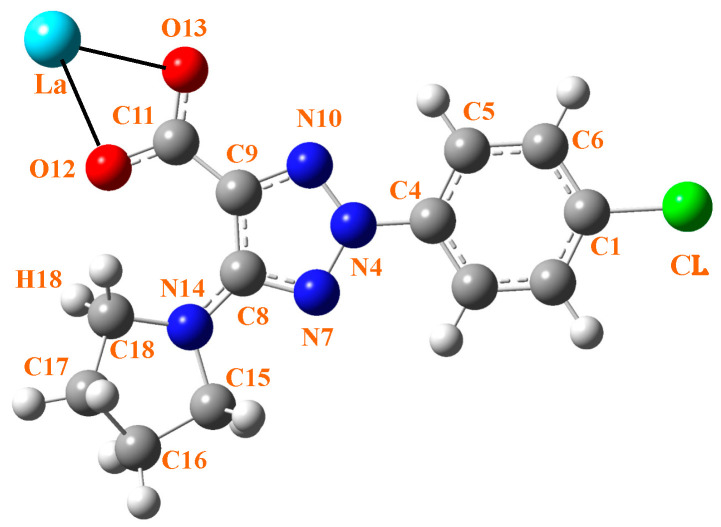
Labeling of the atoms in 2b′ ligand with its bonding to La(III) ion through the carboxylate group.

**Figure 2:**
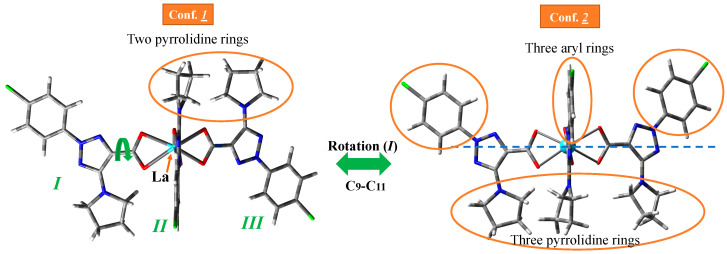
The two possible stable conformers specifically for the La(2b′)_3_ complex by rotation of the ligands. They were the starting point for the detailed conformational and hydration studies and subsequent spectral analyses presented in this manuscript. Although similar conformational studies have been reported for other lanthanide complexes [33], this figure focuses on the fundamental structure relevant to the comprehensive spectroscopic and computational investigations unique to this specific La(III) complex within the current work. The substituents on the same side are circled in orange (Conf. ***2***).

Three ligands must bind to a La(III) ion in coordinated form. Therefore, the ligand must contain a strong accepting group, such as the carboxylate group. In addition, the ligand should possess lipophilic moieties that can imbue the complex with the liposolubility needed to facilitate cell membrane permeation. The aryl and pyrrolidine groups were selected for this purpose. Therefore, the 2-(4-chlorophenyl)-5-(pyrrolidin-1-yl)-2*H*-1,2,3-triazole-4-carboxylate ligand (labeled as 2b′) was synthesized in its acidic and anionic forms [29,30]; see Figure 1. In this complex, the La(III) ion appears coordinated through the carboxylate group (COO^−^) in an almost symmetric three-dimensional system, as shown in Figure 2. This arrangement of the ligands appears to be in accordance with data reported in related complexes with La(III) [10,31].

This newly synthesized complex presented significant pharmacological interest in our previous work [32], which demonstrated significant, concentration-dependent interactions of La(2b′)_3_ with a variety of reactive species-generating model systems (DPPH, ABTS, UV-induced water radiolysis, Fenton reaction, hypochlorite, superoxide). These findings highlight the complex’s promise as a potential antioxidant, antimicrobial, or anticancer agent. Given the significant importance of this complex and the inability to determine its molecular structure through X-ray analysis, the current study aimed to achieve the following objectives: (i) to accurately characterize and assign its whole IR and Raman spectra considering the presence of hydration water molecules in its molecular structure; (ii) to predict its reactivity by the calculation of the highest occupied molecular orbital (HOMO), lowest unoccupied molecular orbital (LUMO), and several global chemical descriptors; (iii) to establish the molecular structure of the type of clusters that can facilitate the synthesis of new ligands with enhanced activity; (iv) to determine several photophysical properties in other lanthanide complexes and compare the results with those of the free ligand, as well as those with the sample in solid and in DMSO solution phase; and (v) to analyze the effect of the lanthanide ion on the molecular structure of the complex through their IR and Raman spectra, along with their properties.

## 2. Results and Discussion

In order to yield an accurate assignment of the whole IR and Raman spectra, it was necessary to optimize, in advance, the molecular structure of the complex in isolated state and, after that, with water molecules to better represent the hydrated complex in the solid-state sample.

### 2.1. Calculated Molecular Structure in the Isolated State

The geometry structure of the La(III) complex was optimized in the isolated state at four DFT levels, namely B3LYP/CEP-4g, CAM-B3LYP/Lanl2dz, M06-2X/Lanl2dz, and M06-2X/Lan2mb. By rotation of the ligands around the C_9_-C_11_ bond, two possible conformers appear stable for this complex; see Figure 2. Conformer ***2*** corresponds to the three ligands on the same side of the structure (the three pyrrolidine rings or the three aryl rings are on the same side), while in conformer ***1***, only two ligands (two pyrrolidine rings) are on the same side, and the third ligand is oriented on the opposite side. Form ***1*** is slightly more stable than form ***2*** at all levels studied, and therefore, it is the only form further analyzed in the present manuscript. The three ligands of the complex are labeled as *I* to *III*.

An almost symmetric arrangement of the ligands around the La(III) ion is obtained for this complex at the CAM-B3LYP/Lanl2dz and M06-2X/Lanl2dz levels (Figure 2), while it is deformed at the slightly lower B3LYP/CEP-4g, and M06-2X/Lan2MB levels with two of the ligands being closer to each other; see Figure S1 (Supplementary Materials). For this reason, the results obtained at these last two levels were considered with caution, and the discussion was mainly focused on the CAM-B3LYP/Lanl2dz and M06-2X/Lanl2dz results. The most characteristic optimized geometrical parameters at these levels are collected in Table 1. For labeling the atoms in the ligands, the notation used is shown in Figure 1, which is in accordance with that previously reported for this ligand [29]. In Table 1, the atoms with (′) correspond to ligand *I*, while the atoms with (″) correspond to ligand *II*. For comparison purposes, the calculated geometrical parameters at the MP2/6-31G(d,p) level in the anionic form of the ligand (2b′) are included in the second column. Close values were obtained by the CAM-B3LYP and M06-2X methods (fourth–fifth columns, respectively), with both including the utilization of the Lanl2dz basis set. The differences are much larger if the comparison is between the values obtained by the Lanl2dz and Lanl2mb basis set (fifth–sixth columns, respectively) using the M06-2X method for both cases. The use of a lower basis set, such as Cep-4g, leads to noticeably larger differences that, in turn, deviate much more significantly from calculations with the MP2 method in the isolated ligand (second column). The intramolecular H-bond O_12_···H_18_ was included in the table because it is present both in the ligand alone and in its hydrated complex. It was also observed in the deformed complexes by using the Cep-4g and Lanl2mb basis sets.

### 2.2. Calculations of the Hydrated Molecular Structure

Due to hydration water molecules present in the solid-state sample, the hydration effects in the isolated molecular structure were calculated following the Discrete Model (DM) [34,35] by including the expected number of explicit water molecules according to the experimental IR spectra. Among the possible procedures to theoretically simulate the hydration, this DM model appears the best, since it provides a whole description of the water molecule net, as well as the specific solute-solvent interactions. Following this model, the first water molecule was introduced in different positions around the La(III) ion due to its high positive charge. The best form (that with the highest total negative energy value) was considered for the next stage, involving the introduction of a second water molecule in different positions. This methodology, called MSM (Modified Scheme of Monosolvation) [36,37], was followed until 10 water molecules were introduced, and it seems to be the best method to obtain the cluster with the global minimum energy (highest negative energy).

**Figure 3:**
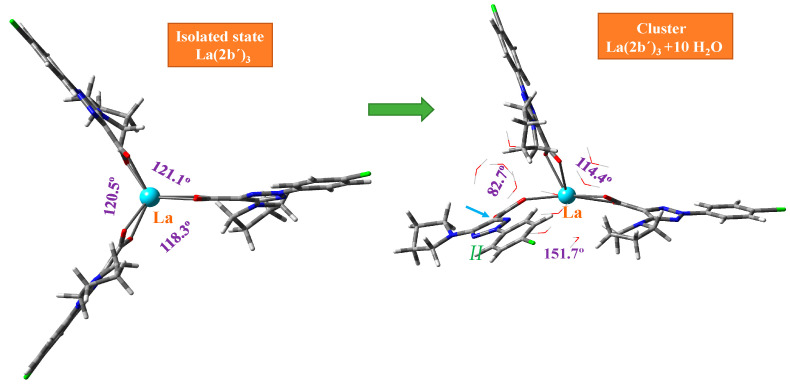
Deformation of the optimized structure of the La(2b′)_3_ complex with hydration at the M06-2X/Lanl2dz level. The angle between the ligands appears in violet. The small blue arrow indicates the break of the La-O12 bond in ligand *II*.

**Figure 4:**
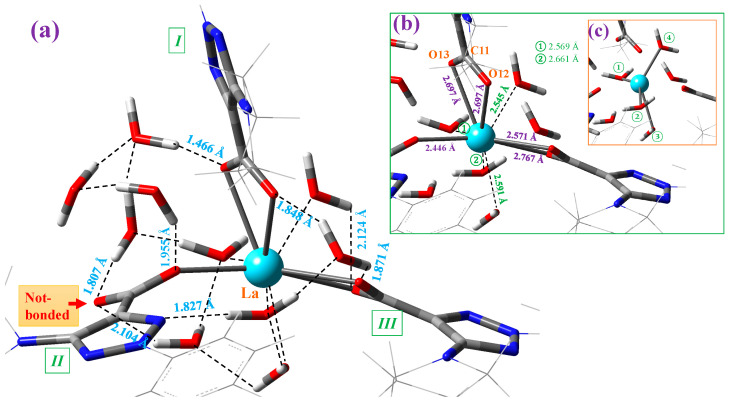
Structure of the cluster with 10 explicit water molecules around the La(III) ion in the La(2b′)_3_ complex optimized at the M06-2X/Lanl2dz level. (**a**) The intermolecular H-bond values between the water molecules and the complex are shown in blue. The remaining interactions in the cluster are also indicated. (**b**) The interaction of four water molecules with the La(III) ion is included in green. The coordination bond values of the carboxylate groups with the La ion are included in violet. (**c**) A clearer plot with the special distribution of the four water molecules distributed around the La ion. These water molecules are indicated by numbers in green circles.

This MSM methodology requires the optimization of a large number of clusters. For simplicity, Figure 3 includes the optimized form at the M06-2X/Lanl2dz level with 10 water molecules, while Figures S2 and S3 include the optimized clusters at the M06-2X/Lanl2mb and CAM-B3LYP/Lanl2dz levels, respectively. The first effect observed with the hydration water is the disruption of the symmetric arrangement of the ligands around the La(III) ion, as can be seen in the isolated state vs. cluster comparison of Figure 3. The second effect observed is the breakage of one of the La-O bonds in ligand *II*, indicated by the blue arrow in Figure 3 and marked in red in an amplification of this figure in Figure 4.

Each of the carboxylate oxygen atoms appear H-bonded to one water molecule, with the exception of the oxygen not bound to the La ion (marked in red in Figure 4a), which is H-bonded to two water molecules. Only one water molecule is H-bonded to N10 nitrogen of ligand *II*. In addition, four water molecules are directly interacting with the La ion; see Figure 4b. The symmetric distribution of these four water molecules can be clearly identified in the marked lines plotted in Figure 4c. Of the ten water molecules of this cluster, only one water molecule is not H-bonded/interacting with the atoms of the La(2b′)_3_ complex. Due to these H-bonds/interactions, the water molecules remain stable as hydration water inside the complex, which is investigated for its IR spectrum in the solid-state sample.

The values of several selected optimized geometric parameters of this cluster are collected in Table 1 and compared to those calculated in the isolated state (second column). The effect of the water molecules on the La(III) ion leads to a lengthening of the La-O bonds—0.05 Å in La-O12 and 0.28 Å in La-O13 with the M06-2X method. This feature is expected to be increased if additional water molecules are added to the model, which can lead to dissociation of the complex and its consequent water solution. This weakening of the La-O bond with hydration leads to a slight stretching of the C=O bonds and an opening of the O-C-O angle. The asymmetry of the La-O12-C vs. La-O13-C angle values is increased with hydration in accordance with a larger difference between the O12 and O13 charges.

At the M06-2X/Lanl2mb level, the optimized cluster with 10 water molecules of Figure S2 shows a noticeably large deformation, with a water proton bonded to a carboxylate oxygen and, therefore, having a OH group free. In addition, two carboxylate oxygens appear not bonded to the La(III) ion, and several water molecules have noticeably longer O-H bond lengths. All these features lead to a predicted IR spectrum noticeably different from the experimental one; see Figure S4. At the CAM-B3LYP/Lanl2dz level, the optimized cluster also shows a large deformation with two carboxylate oxygens not bonded to the La(III) ion, but its predicted IR spectrum can be compared to the experimental one.

### 2.3. Calculation of Atomic Charges and Molecular Properties

The interaction of water molecules with the La(III) ion noticeably reduces its positive charge, which affects charge distribution in the neighbor atoms; see Table 2. This distribution is also affected by the H-bonds of water molecules with the oxygen atoms and, therefore, by the C9 and C11 carbon atoms, the charges of which change noticeably. For example, using M06-2X in C11, the charge changes from 1.821*e* in isolated state to 1.635*e* in hydrated form, and using M06-2X in C9 changes the charge from −0.835*e* to −0.614*e* in the hydrated form. Since the oxygen atoms bear the highest negative charge, they continue being the most reactive, as well as the N14 atom, ands therefore, they are expected to play a key role in the antioxidant and pharmacological activity of this compound. In the triazole ring, only N4 has a significant negative charge, −0.660*e* in the hydrated form.

Several thermodynamic parameters, rotational constants, and dipole moments were also calculated, and they are included in Table 3. Because of the symmetry of the complex at the M06-2X/Lanl2dz and CAM-B3LYP/Lanl2dz levels, the rotational constant values in the three directions (A, B, C) have almost the same value. They differ, however, in the other two DFT levels. One value is larger (A-axis), corresponding to the ligand noted as *I*. The two values corresponding to ligands *II* and *III* are lower (B- and C-axes). The C_v_ and entropy (S) values were close to M06-2X and lower than those calculated by B3LYP. This feature may be due to the lower symmetry of the complex obtained by B3LYP. The calculated dipole moment value indicates that this complex has slight water solubility, which could facilitate its biomedical use.

**Figure 5:**
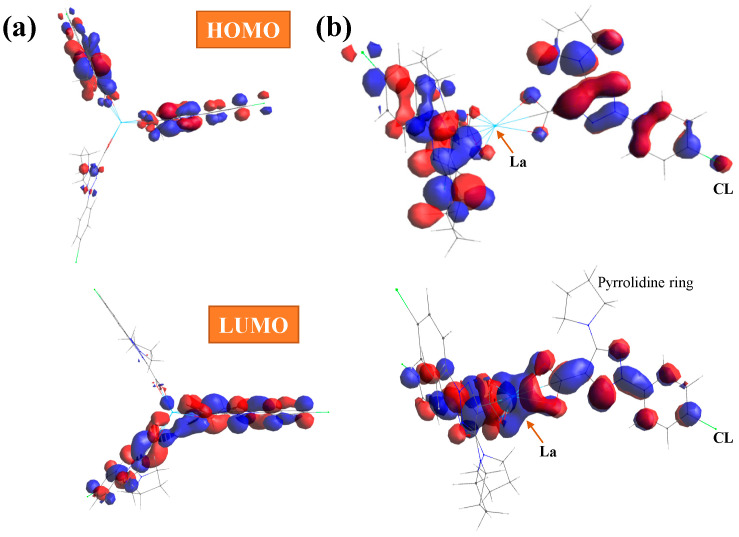
(**a**) Front and (**b**) lateral views of the HOMO and LUMO plots in the La(2b′)_3_ complex calculated at the CAM-B3LYP/Lanl2dz level. The red and blue colors correspond to the positive and negative phase, respectively.

With the HOMO (highest occupied molecular orbital) and LUMO (lowest unoccupied molecular orbital) values, the global chemical reactivity descriptors were calculated [38,39] to better understand the reactivity and stability of the La(2b′)_3_ complex under study. In the present complex, the HOMO orbital shows the charge density localized over the triazole ring and the neighbor carbons atoms, while the LUMO is defined by charge distribution mainly on the oxygen atoms and few aryl carbon atoms, which appear available to accept electrons from the triazole ring; see Figure 5. Charge distribution is also observed on the triazole ring and the carbon atoms of the phenyl ring, except for C_2_ and C_6_, but it is very small on the chlorine atom, and it is not observed on the pyrrolidine ring atoms and La ion.

The energy gap (E_g_) between the HOMO and LUMO frontier orbitals is a significant characteristic of molecules and helps characterize their chemical reactivity and kinetic stability. A high E_g_ in a molecule indicates that it is less polarizable. It is generally associated with low chemical reactivity and high kinetic stability. Therefore, our low E_g_ values in the La(2b′)_3_ complex calculated by all three DFT methods indicate large chemical reactivity and small excitation energies to the manifold of excited states. The hydration water affects the energy of the orbitals only very slightly, and thus, only a little reduction in the negative HOMO energy is observed, with E_g_ being 0.011 eV smaller.

The calculated value for the ionization potential (IP) is somewhat low, in accordance with the large reactivity of the complex. The electron affinity (EA) is remarkably lower than the IP and positive, with the exception of the M06-2X/Lanl2mb calculation. The electronegativity (χ) is low, in accordance with a neutral system. Chemical hardness (η) and global softness (S) express the resistance of a system to a change in its number of electrons. When η is weak, the molecule is called soft, and it has a small HOMO–LUMO gap, and when it is high, the molecule is called hard. Our low values correspond to a soft molecule with a small gap and an electron density that can change easily.

### 2.4. Vibrational Analysis

For the identification and characterization of the synthesized La(2b′)_3_ coordination complex, a detailed analysis of the calculated and experimental spectra was carried out. For this purpose, the first step was to correct (to scale) the theoretical calculated spectra by applying several scaling procedures [40,41]. Further comparison of these scaled spectra in the whole 3750–50 cm^−1^ range at all DFT levels used with the experimental ones was carried out. Consequently, the theoretical level that has the best agreement with the experimental spectrum can be determined, and based on this selection, the assignment of the experimental bands can be carried out. Under this methodology, the scaled IR and Raman spectra in the isolated state at the M06-2X/Lanl2dz, M06-2X/Lanl2mb, B3LYP/Cep-4g, and CAM-B3LYP/Lanl2dz levels were used. In an initial comparison to the experimental findings, the following observations were made:

- A very broad band, centered at ca. 3400 cm^−1^, is observed in the experimental IR spectrum, which can only correspond to the O-H stretching ν(O-H) mode of hydration water strongly H-bonded to the ligands in the La(2b′)_3_ complex system. Due to the spatial arrangement of the ligands in the complex, many holes appear in the structure, which can be occupied by water molecules, associated with the synthesis of the complex. Due to the large negative charge around the three carboxylate groups, water molecules can be H-bonded through their hydrogen atoms. Moreover, this band does not appear in the Raman spectrum, as expected.
- A broad and very strong band centered at 1577.8 cm^−1^ in the experimental IR spectrum, in which the in-plane bending δ(O-H) mode of these hydrated water molecules contributes to its broadness.

Due to the presence of hydration water molecules in the solid-state sample, a previous optimization with different number of water molecules and in different positions around the La(III) ion and the carboxylate group was carried out, and the scaled spectra were obtained. Since the Raman spectrum does not include bands corresponding to the water molecules, it was studied first. Unfortunately, the experimental spectrum shows large background noise that impedes the detection of all weak bands. However, in the isolated state, the strongest bands in the theoretical scaled spectra at the M06-2X/Lanl2dz, M06-2X/Lanl2mb, and CAM-B3LYP/Lanl2dz levels are in very good agreement with the experimental one, which clearly characterizes and confirms the synthesized La(2b′)_3_ coordination complex. The only difference appears in the experimental Raman line observed at 72 cm^−1^, which was not reproduced in the theoretical scaled ones.

**Figure 6:**
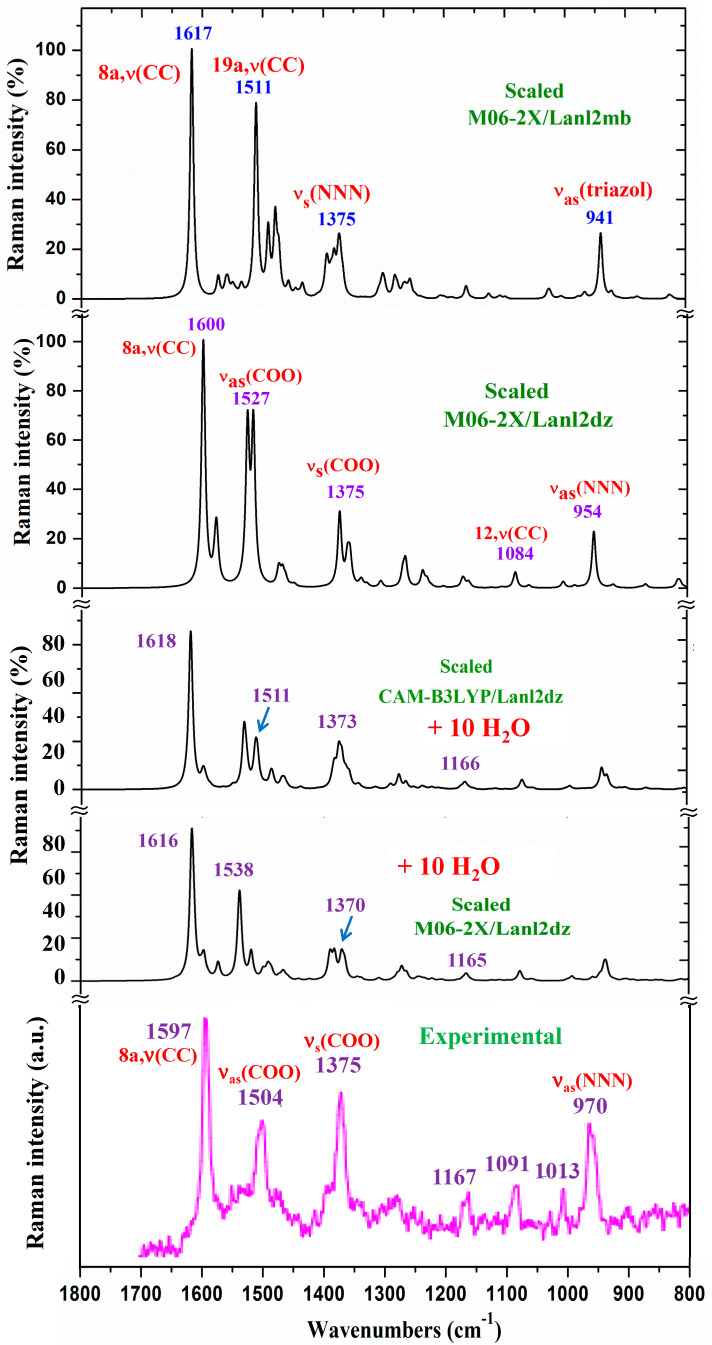
Comparison of the scaled Raman spectrum at the M06-2X/Lanl2dz level and that scaled, following the Polynomic Scaling Equation (PSE) procedure, with the experimental one in the 1800–800 cm^−1^ range.

Due to the lower quality of this experimental Raman spectrum, Figure 6 shows only the spectra comparison in the 1800–800 cm^−1^ range. The theoretical comparison in other spectral ranges is included as part of the Supplementary Materials. Therefore, the whole Raman spectra is included in Figure S4, the region from 3400 to 2600 cm^−1^ is shown in Figure S5, the section from 1800 to 1000 cm^−1^ is shown in Figure S6, and the spectrum from 1000 to 50 cm^−1^ is shown in Figure S7. The assignment of the strong and characteristic Raman lines is included in these figures. The scaled spectrum at the B3LYP/Cep-4g level clearly differs from the experimental one and, therefore, was only included as part of the Supplementary Materials. Due to the close spectra of the M06-2X/Lanl2dz and CAM-B3LYP/Lanl2dz levels, the latter were omitted from the figures. A slight improvement in the agreement of the scaled spectrum vs. experimental ones was obtained with the scaled spectra with 10 water molecules. For simplicity, only those determined at the M06-2X/Lanl2dz and CAM-B3LYP/Lanl2dz levels were included in Figure 6.

**Figure 7:**
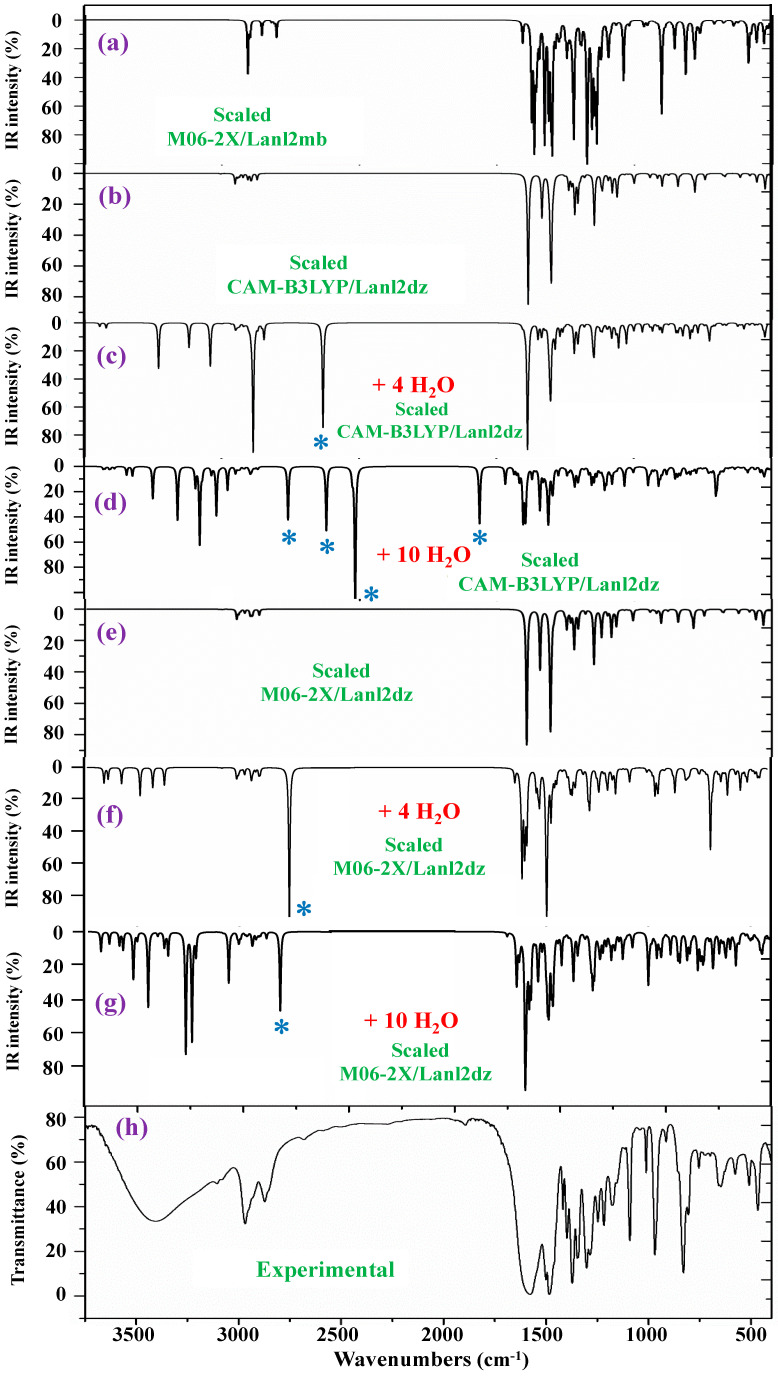
Comparison of the scaled IR spectra in the isolated state and with hydration water molecules at different DFT levels with the experimental ones. * ν(OH) in strong H-bonded water molecules.

The IR spectra were also studied. They were noticeably more complex due to the presence of water molecule bands. In this case, a comprehensive comparison of all scaled spectra obtained was performed, and a resume of them with seven selected scaled spectra and the corresponding experimental one in the 3750–400 cm^−1^ range is included in Figure 7. This comparison includes the scaled spectra with a low number of hydrated water molecules (4 molecules) and a large number of them (10 molecules). Therefore, the differences can be better observed. All these spectra are plotted at the same scale; thus, comparisons between each other and with the experimental one can be easily made. Other scaled spectra with larger differences when comparing with the experimental one are included in Figure S8. In a general comparison of these scaled spectra, it was noted that most of the calculated normal modes appear in their expected ranges. This feature, together with the fact that most of the scaled strongest vibrations achieved by the M06-2X and CAM-B3LYP methods appear close in wavenumber to the experimental ones, confirm the scaling carried out on the calculated wavenumbers, as well as the methods used. Therefore, the further assignments of the bands could be considered generally correct. Because the IR spectrum at the B3LYP/Cep-4g level was the worst, their values were omitted from the discussion. After a general comparison of the spectra, the following observations were made:

- Very obvious similarity between the scaled spectra in the isolated state at the M06-2X/Lanl2dz and CAM-B3LYP/Lanl2dz levels was observed, which could be due to the symmetric and better optimized structure obtained at these levels. However, the spectrum at the M06-2X/Lanl2mb level has the best correlation with the experimental one, perhaps due to its deformed

Due to its deformed and more packing form, which is expected to be reflected in the crystal of the solid-state sample. The spectrum at the B3LYP/Cep-4g level differs remarkably, and therefore, it was included in Figure S8.

**Figure 8:**
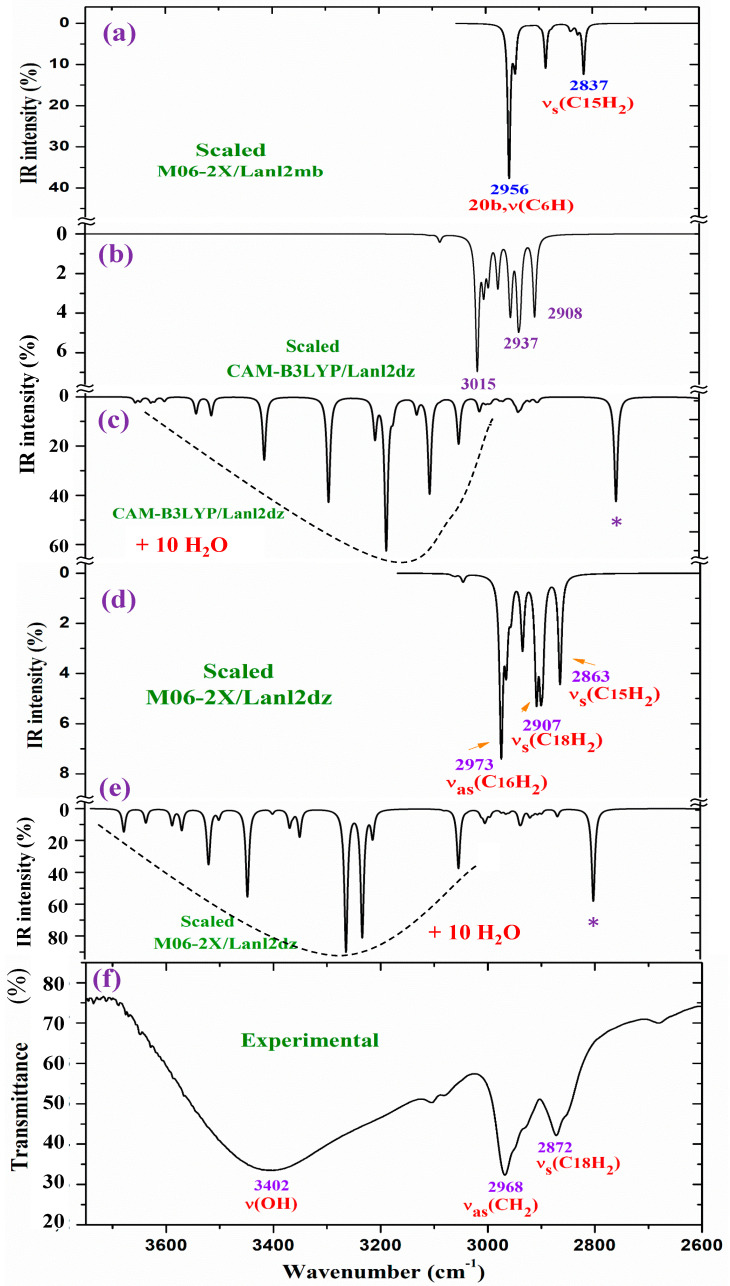
Results for the 3750–2600 cm^−1^ range of the scaled IR spectrum in the isolated La(2b′)_3_ complex: (**a**) M06-2X/Lanl2mb level, (**b**) CAM-B3LYP/Lanl2dz level, and (**c**) results for the scaled spectrum of the La(2b′)_3_ + 10 H_2_O cluster at the CAM-B3LYP/Lanl2dz level, (**d**) in the isolated complex at M06-2X/Lanl2dz level, (**e**) in the La(2b′)_3_ + 10 H_2_O cluster at M06-2X/Lanl2dz level, and (**f**) the experimental spectrum for the solid-state sample. * ν(OH) in strong H-bonded water molecules.

- The coordination of the 2b′ ligands to the lanthanum ion noticeably changes the IR and Raman spectra, and therefore, they seem different from those obtained with the 2b′ ligand molecule alone in its anionic form [29].
- The scaled spectra with four hydration water molecules do not appropriately reproduce the broad band centered at 3401.6 cm^−1^ (Table 4) observed in the experimental spectrum. In addition, a very strong band, with the highest IR intensity of the spectrum appears at 2927 cm^−1^ by CAM-B3LYP without correspondence to the experimental spectrum. This very strong band, assigned as ν_as_(O-H), corresponds to a water molecule directly bonded to the La ion, but it seems that it does not occur in the synthesized solid-state sample.
- The scaled spectra with 10 hydration water molecules at the M06-2X/Lanl2dz and CAM-B3LYP/Lanl2dz levels somewhat reproduce the broad experimental band centered at 3401.6 cm^−1^, although it is reproduced with an IR intensity slightly higher than that observed in the experimental spectrum and at ca. 100–200 cm^−1^ to lower wavenumbers. For clarity, this broad experimental band was reproduced theoretically with dotted lines involving the scaled ν(O-H) stretching bands of the water molecules; see Figure 8. It was reproduced slightly better by the M06-2X method than by CAM-B3LYP, with the band center being at a higher wavenumber (ca. 100 cm^−1^) than by CAM-B3LYP and closer to the experimental band. The scaled spectra with seven or eight hydration water molecules appear the best. The M06-2X/Lanl2mb level dramatically fails in the calculation of the hydrated form cluster, and its spectrum shows very strong ν(O-H) stretching bands corresponding to the water molecules and with remarkably higher intensity compared to the remaining bands of the spectrum; therefore, it was included in Figure S8b.
- In our hydration model, water molecules are directly H-bonded to the carboxylate group or strongly interacting with the La ion. This causes them to be slightly deformed, with O-H bond lengthening, and therefore, the ν(O-H) stretching vibrations strongly shift to lower wavenumbers. The calculated bands of these water molecules are indicated by a blue (*) symbol in Figure 7. Since they do not have correspondence to the experimental spectra, it means that these water molecules should be present around other active atoms of the complex with weaker H-bonds. This explanation is also supported by the fact that the experimental broad band, corresponding to ν(O-H)_H2O_, appears centered at 3406 cm^−1^, while the scaled main bands of this mode appear around 3250 cm^−1^ in the 10-hydration-water-molecule spectra.
- The scaled spectrum with 10 water molecules by CAM-B3LYP/Lanl2dz shows much more water vibrational bands than that by M06-2X/Lanl2dz, which is due to a noticeably larger effect of the water molecules by CAM-B3LYP than by M06-2X on the COO group, with the breaking of two O-La bonds, as described in Section 2.2.

**Figure 9:**
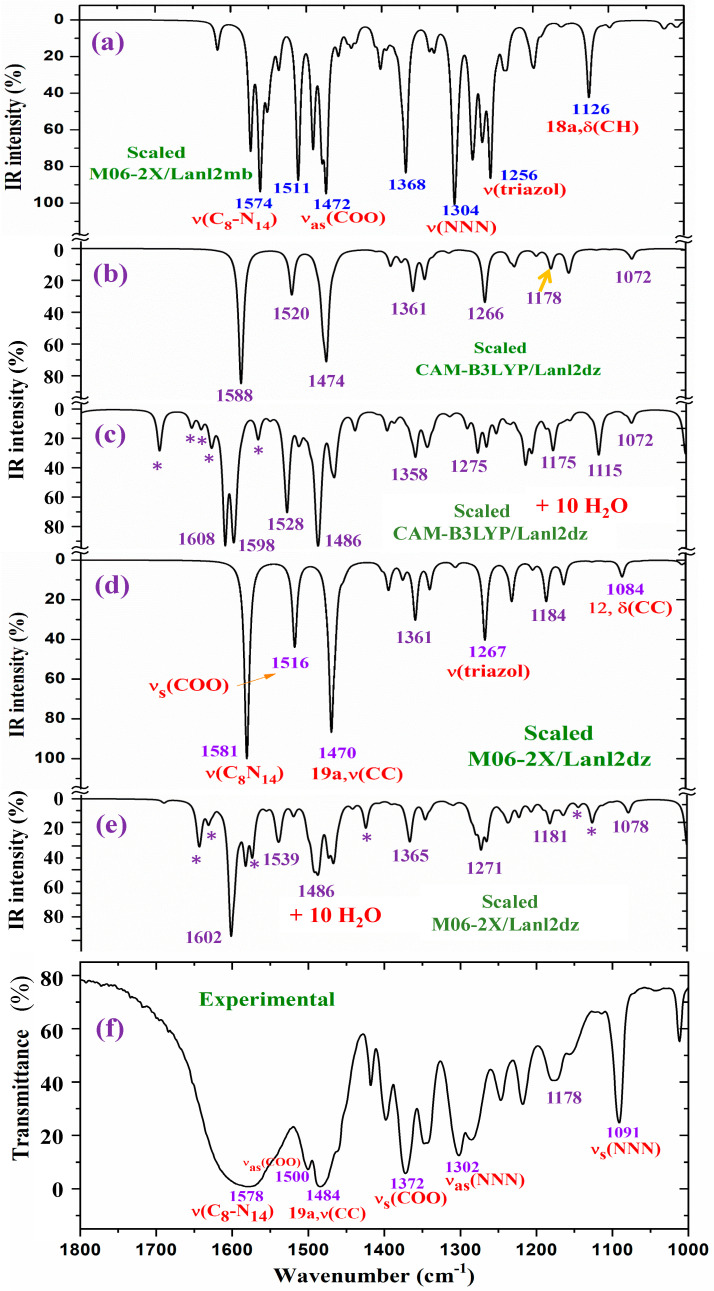
Results for the 1800–1000 cm^−1^ range of the scaled IR spectrum in the isolated La(2b′)_3_ complex: (**a**) M06-2X/Lanl2mb level, (**b**) CAM-B3LYP/Lanl2dz level, and (**d**) M06-2X/Lanl2dz level. Results for the scaled spectrum of the La(2b′)_3_ + 10 H_2_O cluster: (**c**) CAM-B3LYP/Lanl2dz and (**e**) M06-2X/Lanl2dz levels. (**f**) The experimental results for the solid-state sample. * ν(OH) in strong H-bonded water molecules.

The vibrational bands of these stronger H-bonded water molecules can appear along the 4000–400 cm^−1^ spectral range, which largely complicates the spectrum. Therefore, all of them were removed from the spectra through an option provided in the Gaussian-16 program package. This way, the scaled spectral bands obtained can be better related to the experimental ones. This difference can be clearly observed in the three ranges in which the IR spectrum has been divided: 3750–2600, 1800–1000, and 1000–400 cm^−1^ (see Figures S9, S10, and S11, respectively). For simplicity, only the difference obtained in the spectra by M06-2X/Lanl2dz was included in these figures, and we omitted those by CAM-B3LYP due to the more disruptive effect of the water molecules in the cluster. Figure S9 shows a comparison between the scaled spectrum in the isolated state and that obtained with 10 hydrated water molecules (+10 H_2_O), as well as that obtained with the experimental one. The symbol (#) in Figure S9b indicates the spectrum obtained with the water molecule bands removed from it. Therefore, the intensity of the C-H stretching bands can be increased and easily compared to the experimental ones. These C-H stretching bands appear with weak-very weak IR intensity in the spectrum of Figure S9c, and they are hidden by the strong-very strong water molecule bands. Figure S10 shows the same comparison but in the 1800–1000 cm^−1^ range. Several δ(O-H) bending vibrational bands corresponding to the water molecules can be observed in Figure S10c compared to Figure S10b, denoted by the symbol (*). Those in the 1650–1550 cm^−1^ range can contribute to the broadening of the experimental band centered at 1577.8 cm^−1^. Water bands in this region have also been determined by CAM-B3lYP; see Figure 9c. After comparing Figure S11b vs. S11c, the largest difference can be observed in the 1000–400 cm^−1^ range, which has many water bands.

**Table 4:** Calculated, scaled, and experimental wavenumbers (ν, cm^−1^) in the La(2b’)_3_ complex. Relative infrared intensity (A) in %, relative Raman intensity (S) in %, and Raman depolarization ratios for plane (DP) and unpolarized incident light (DU). For each vibration of the tetramer, the wavenumber with the highest IR intensity is indicated in bold, and that with the highest Raman intensity is indicated in italic type. Only the relative IR and Raman intensities are shown for these wavenumbers. DP and DU values were derived from the most intense Raman line.

**Calculated at M06-2X/Lanl2dz**					**Scaled**	**Experimental**		**Characterization**
**ν**	**A**	**S**	**DP**	**DU**		**IR**	**Raman**	
						3401.6 br, s		ν(O-H) H_2_O bonded
3188, 3187, ***3187***	6	3	0.72	0.84	2973	2968.1 s		ν_as_(C-H) in C16H_2_, C17H_2_ (100)
***3116***, 3116, 3114	4	4	0.03	0.06	2907	2872.2 m		ν_s_(C-H) in C18H_2_ in pyrrolidine (100)
1687, 1687, *1686*	0	55	0.39	0.56	1600		1597 vs	8a, ν(C=C) in aryl (89)
**1666**, *1663*, 1662	100	24	0.06	0.12	**1581**, *1578*	1577.8 br, vs		ν(C8-N14) (73) + ν_s_(N7CC) (15)
*1607*, 1596, **1596**	46	100	0.08	0.15	*1527*, **1516**	1500.2 s	1504 s	ν_as_(COO) (49) + ν(C9-C11)
1549, *1544*, **1543**	83	5	0.75	0.86	1474, ***1470***	1484.5 vs		19a, ν(CC)(35) + δ(CH) in pyrrolidine (15)
**1461**, 1455, *1454*	28	0	0.75	0.86	**1393**, 1388	1398.1 m		ν_as_(CCOO) + ν_s_(NNN) + ν(C-N)
1441, *1440*, **1440**	8	21	0.32	0.48	1375	1372.3 vs	1375 vs	ν_s_(COO) + ν_s_(NNN) + δ_s_(CC,CH)
1404, **1402**, *1401*	14	2	0.75	0.85	1341, **1340**	1347.5 s		δ_s_(C-H) in pyrrolidine (30)
1363, 1363, 1363	2	0	0.38	0.55	1304	1302.0 vs		Γ((pyrrolidine)
1326, ***1322***, 1321	42	12	0.75	0.86	1270, ***1267***	1285.5 s		ν(NN,CN) + γ_as_(CC,CH) in pyrrolidine
*1291*, **1291**, 1290	2	4	0.65	0.79	1238	1246.8 m		ν_as_(NN,CN)+3,δ(CH) in aryl+δ(pyrrolidine)
1285, ***1283***, 1283	22	2	0.75	0.86	1232, **1230**	1218.0 m		γ_as_(C-H) out-of-phase in pyrrolidine
1235, **1233**, *1232*	22	0	0.75	0.86	1186, **1184**	1178.2 m		γ_as_(C-H) in pyrrolidine
1123, 1123, 1123	8	5	0.02	0.04	1084	1091.2 vs	1091 m	12, δ(CC,CH) in aryl (96)
*1037*, 1037, **1036**	2	1	0.22	0.36	1005	1011.8 m	1013 w	18a, δ(CC, CH) in aryl (98)
981, 981, 981	10	17	0.07	0.14	954	969.0 vs	970 s	ν_as_(NNN) + 18a, δ(CC,CH) in aryl
*830*, **828**, 827	4	3	0.04	0.07	*816*, **814**	829.7 vs		γ_s_(COO) + γ(triazol)
*826*, **819**, 818	16	3	0.04	0.07	812, **805**	805.4 m		δ_as_(COO) + γ(C9-C11)
675, *675*, **674**	2	0	0.73	0.85	674	654 m		γ_s_(NNN) + δ_as_(COO)
**668**, *668*, 667	2	0	0.73	0.84	668	647.1 m		((triazol) (65) + δ_as_(COO) (18)
511, ***507***, 507	8	0	0.74	0.85	524, **520**	508.9 m		δ_as_(NNN) +δ(CCL) +((CC) aryl +δ_as_(COO)
*472*, **469**, 468	12	1	0.34	0.50	489, **486**	466.5 m		δ_as_(COO) in phase + 6b, δ(CCC) in aryl +La
**Calculated at M06-2X/Lanl2MB**					**Scaled**	**Experimental**		**Characterization**
**ν**	**A**		**S**			**IR**	**Raman**	
			--		--	3401.6 br, s		ν(O-H) H_2_O bonded
3553, 3553, 3553	26		3		2956	2968.1 s		20b, ν(C6-H) in aryl (100)
3488, 3468, ***3453***	14		16		2911, ***2892***	2872.2 m		ν_as_(C-H) in C15H_2_ pyrrolidine (100)
1769, 1769, 1769	9		97		1617		1597 vs	8a, ν(C=C) in aryl (93)
***1716***, 1701, 1700	83		22		***1574***, 1560	1577.8 br, vs		ν(C8-N14) + ν_s_(N7CC)
1640, 1640, 1639	55		100		1511	1500.2 s	1504 s	19a, ν(CC,CH) + ν(C4-N4) +ν(C9-C11)
1616, *1602*, **1595**	100		80		1491, *1477*, **1472**	1484.5 vs		ν_as_(COO) + ν_s_(CCN)triazol+ν(C9-C11) + 19a,ν(CC)
1501, ***1500***, 1498	7		16		***1395***, 1392	1398.1 m		δ_s_(C-H) in pyrrolidine + ν_s_(NNN)
1484, ***1477***, 1475	19		33		1380, ***1375***, 1373		1375 vs	ν_s_(NNN) + ν_s_(COO) + γ_s_(C-H) in pyrrolidine
*1474*, **1469**, 1468	71		18		*1372*, **1368**	1372.3 vs		ν_s_(COO) + ν_s_(NNN) + δ_s_(CC,CH) + 19a, ν(CC)
***1433***, 1425, 1424	15		1		***1337***, 1329	1347.5 s		Γ((C-H) in pyrrolidine
1399, **1394**, *1390*	94		16		1309, **1304**, *1301*	1302.0 vs		ν_as_(NNN)(36) + δ(C-H) in pyrrolidine (30)
*1367*, **1365**, 1362	50		16		*1281*, **1279**, 1276	1285.5 s		δ_as_(C-H) in pyrrolidine + ν(triazol)
1339, ***1337***, 1336	82		8		1258, ***1256***	1246.8 m		ν(triazol) + 3, δ(C-H) + δ(C-H) in pyrrolidine
1312, 1311, 1311	17		0		1234	1218.0 m		14,ν(CC) in aryl + ν_as_(NN) + δ(C-H) pyrrolidine
**1269**, *1259*, 1256	22		1		**1198**, *1189*, 1186	1178.2 m		ν(triazol) + γ_as_(C-H) in pyrrolidine + ν_s_(COO)
1155, 1154, **1153**	3		1		1099, **1097**	1091.2 vs	1091 m	ν_s_(NNN) + 18a, δ(C-H) in aryl
**1054**, *1050*, 1050	3		1		**1011**, *1007*	1011.8 m	1013 w	ν_as_(triazol) + δ(CC,CH) pyrrolidine + δ_s_(COO)
974, 973, 973	36		24		941	969.0 vs	970 s	ν_as_(NNN, CC) in triazol + 18a, δ(C-H) in aryl
*846*, **842**, 840	36		3		*827*, **824**, 822	829.7 vs		δ_as_(COO) + γ_as_(C-H) pyrrolidine + ν(triazol)
799, ***792***, 789	26		1		785, ***779***, 777	805.4 m		δ_as_(COO) + γ_as_(C-H) pyrrolidine + ν(triazol)
658, 658, 658	3		1		658	654 m		6a, δ(CC) in aryl
**638**, *636*, 633	2		0		**640**, *638*, 635	647.1 m		Γ((triazol) + γ_as_(COO) + γ(C-H) pyrrolidine
**504**, *500*, 489	32		3		**518**, *514,* 503	508.9 m		δ_as_(COO)phase + δ_as_(triazol) + ν(CCL) + 6b,δ(CCC)
*462*, **460**, 457	13		1		*479*, **477**, 474	466.5 m		ν(CCL) + δ_as_(COO) + δ(NNN) + 6b,δ(CCC)

According to these observations, a resume of the main experimental and calculated wavenumbers in the isolated state at the M06-2X/Lanl2mb and M06-2X/Lanl2dz levels is shown in Table 4, where only the wavenumbers with high IR or Raman intensity are included. The whole spectra assignment is included in Tables S1–S3 (Supplementary Materials), corresponding to the M06-2X/Lanl2dz, M06-2X/Lanl2mb, and B3LYP/Cep-4g levels, respectively. All data correspond to the most stable conformer (conformer ***1***), with the ligands′ orientation being as it is plotted in Figure 2. The data and spectra at the CAM-B3LYP/Lanl2dz level were omitted due to their similarity with those at M06-2X/Lanl2dz.

The first column of Table 4 lists the calculated wavenumbers at the M06-2X/Lanl2dz (first set of values) and M06-2X/Lanl2mb (second set of values) levels. Three values appear for each vibrational mode corresponding to the three ligands of the complex. Of these three values, the one with higher calculated IR intensity is shown in bold, while the one with higher Raman intensity is shown in italics. This notation is omitted if these three values are the same, or show almost null IR or Raman intensity. The second and third columns present the relative IR and Raman intensities (%) corresponding to the wavenumber typed in bold and in italics, respectively. The relative intensities are obtained by normalizing each calculated value to the intensity of the strongest one in the spectrum. The values scaled by the Linear Scaling Equation (LSE) or Polynomic Scaling Equation (PSE) procedures are included in another column, where the notation used for the values is the same as that for the first column. In the next two columns, the experimental IR and Raman values observed in the spectra are listed. The last column corresponds to the main characterization of the vibrations at the M06-2X/Lanl2mb or M06-2X/Lanl2dz levels. In few cases, the % contribution of the different modes to a computed value Potential Electron Distribution (PEDs) is included. The ring mode number corresponds to Wilson′s notation [42]. The values calculated at the B3LYP/Cep-4g level have only been included in Table S3 due to their large differences.

**Figure 10:**
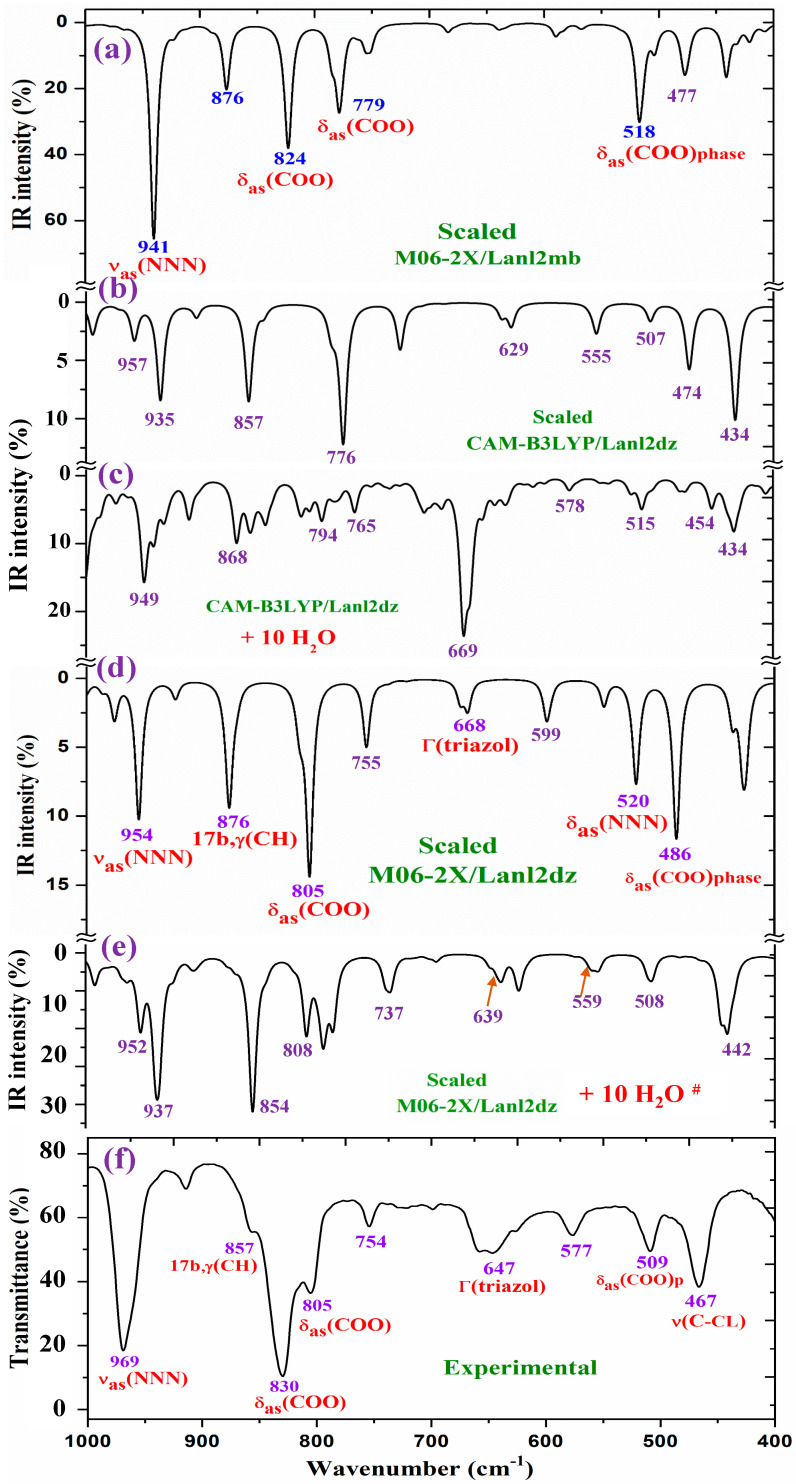
Results for the 1000–400 cm^−1^ range of the scaled IR spectrum in the isolated La(2b′)_3_ complex: (**a**) M06-2X/Lanl2mb level, (**b**) CAM-B3LYP/Lanl2dz level, and (**d**) M06-2X/Lanl2dz level. Results for the scaled spectrum of the La(2b′)_3_ + 10 H_2_O cluster: (**c**) CAM-B3LYP/Lanl2dz and (**e**) M06-2X/Lanl2dz levels. The symbol (#) on H_2_O represents the removal of the water bands. Spectrum (**f**) corresponds to the experimental results for the solid-state sample.

For a detailed and better analysis of the different experimental and scaled vibrational wavenumbers, the spectra are divided into three ranges, which, in the IR spectra, are from 3750 to 2600 cm^−1^ (Figure 8), from 1800 to 1000 cm^−1^ (Figure 9), and from 1000 to 400 cm^−1^ (Figure 10). In a general analysis of these spectra, the following observations were made:

The experimental ν(CH_2_) stretching bands at 2968.1 and 2872.2 cm^−1^ in Figure 8 appear with remarkably higher intensity than predicted theoretically at the CAM-B3LYP/Lanl2dz and M06-2X/Lanl2dz levels in the isolated state and in the cluster with 10 water molecules. Only the bands calculated in the isolated state in the spectrum at the M06-2X/Lanl2mb level have an intensity and wavenumber close to the experimental one. This can be only explained by the packing deformed structure optimized at this level (Figure S1b), with two of the ligands interacting closely, especially their pyrrolidine rings. This interaction may modify the dipole moment and, therefore, the stretching IR intensity in the CH_2_ groups of the pyrrolidine rings. The packing structure obtained in the isolated state at the M06-2X/Lanl2mb level disappears in its hydrated form (Figure S2) due to the introduction of the water molecules between the ligands.

The broad experimental band at 1577.8 cm^−1^ in Figure 9 can be reproduced well by the inclusion of the water bands, in contrast to the narrow band obtained in the isolated state. The very strong experimental bands at 1484.5 and 1372.3 cm^−1^ are also well reproduced with the hydration. For simplicity, the assignment of these bands and others according to the hydration model is included in a resume in Table 5.

The wavenumber of the very strong experimental bands at 969.0 and 829.7 cm^−1^ was reproduced well at the M06-2X/Lanl2mb level (Figure 10a). However, calculations in the isolated state at the CAM-B3LYP/Lanl2dz and M06-2X/Lanl2dz levels fail in their determination, especially the band at 829.7 cm^−1^, somewhat overpredicting at 857 cm^−1^ (by CAM-B3LYP) and at 876 cm^−1^ (by M06-2X/Lanl2dz). The packing interaction of two ligands appears to have influence in these vibrational modes. However, the remaining experimental bands in this figure appear to be well reproduced in the optimized clusters; see Table 5.

The assignment and further discussion of the bands were carried out only under the following modes: (i) the COO group modes, (ii) the triazole ring modes, and (iii) the aryl ring modes. The remaining normal modes, Table 4 and Tables S1–S3, are self-explanatory.

#### 2.4.1. The Carboxylate COO Group Modes

In the isolated state of the 2b′ ligand, the ν_as_(COO) stretching mode was predicted (scaled) with very strong IR intensity at 1710 cm^−1^ [29]. However, in the La(2b′)_3_ complex, it is expected to be significantly red shifted to lower wavenumbers because of the noticeable lengthening of the CO bonds to form the six O-La bonds. At the M06-2X/Lanl2mb level, this stretching mode was clearly identified in the calculated wavenumber, with the strongest IR intensity being found at 1595 cm^−1^ (scaled at 1472 cm^−1^), in very good agreement with the very strong experimental IR band at 1484.5 cm^−1^. However, at the M06-2X/Lanl2dz level, the symmetric ν_s_(COO) mode was clearly characterized first at higher wavenumbers, compared to the asymmetric mode. It was at 1596 cm^−1^ (scaled at 1516 cm^−1^), with strong IR intensity, and at 1607 cm^−1^ (scaled at 1527 cm^−1^) with very strong Raman intensity. Both scaled values were in good concordance with the strong experimental IR band at 1500.2 cm^−1^ and the strong Raman line at 1504 cm^−1^. However, this assignment differs from that found at the M06-2X/Lanl2mb level.

The scaled spectrum at the M06-2X/Lanl2mb level appears to be a slightly better fit with the experimental ones (Figure 9). The asymmetric stretching vibrations for the carboxylate group (COO^−^) are reported to exhibit strong IR absorption near 1600–1560 cm^−1^ for solid-state samples [43,44] and at higher wavenumbers than the symmetric ones. For these reasons, we assign the experimental IR spectrum mainly to that calculated at the M06-2X/Lanl2mb level.

The symmetric ν_s_(COO) stretching mode appears scaled at the M06-2X/Lanl2mb level at 1368 cm^−1^ (IR) and at 1372 cm^−1^ (Raman), with strong intensity, in excellent agreement with the very strong experimental IR band at 1372.3 cm^−1^ and the Raman line at 1375 cm^−1^. These values are also in accordance with the ones reported in solid-state samples of related compounds [43,44], near the 1420–1400 cm^−1^ range for this symmetric stretching mode. At the M06-2X/Lanl2dz level, the asymmetric (instead of the symmetric) mode was the one characterized by the wavenumber calculated at 1440 cm^−1^ (scaled at 1375 cm^−1^), also in good agreement with the experimental spectra at 1372.3 cm^−1^ (IR) and 1375 cm^−1^ (Raman).

#### 2.4.2. The Triazole Ring Modes

The characteristic normal modes of the 1,2,3-triazole ring have been reported [45,46,47], and they are in accordance with our calculations. For simplicity, the discussion was only focused on the assignment of the strongest bands.

*NNN modes:* The ν_s_(NNN) stretching appears strongly coupled with the ν_s_(COO) mode, as well as with other ring modes. At the M06-2X/Lanl2mb level, the highest contribution of this mode was identified in the scaled wavenumber at 1375 cm^−1^, with strong Raman intensity, in excellent agreement with the very strong Raman line at the same value, 1375 cm^−1^. A large contribution of this mode was also observed in the scaled wavenumber at 1368 cm^−1^, whose major contribution corresponds to the ν_s_(COO) stretching mode. At the M06-2X/Lanl2dz level, this mode was identified at the same scaled wavenumber but strongly coupled with the ν_as_(COO) mode. In the isolated state of the 2b′ ligand, it was scaled at 1360 cm^−1^ and related to the experimental IR band at 1340.5 cm^−1^ [29]. Another triazole ring stretching mode that has a symmetric character and is strongly coupled with an aryl ring mode was identified in the scaled wavenumber at 1097 cm^−1^ and showed good agreement with the very strong experimental IR band at 1091.2 cm^−1^ and the Raman line at 1091 cm^−1^.

The ν_as_(NNN) stretching mode appears characterized at the M06-2X/Lanl2mb level in the scaled wavenumber with very strong IR intensity at 1304 cm^−1^, also in excellent agreement with the very strong experimental IR band at 1302.0 cm^−1^.

*C8-N14 modes:* The stretching mode is predicted at the M06-2X/Lanl2mb level with very high IR intensity and medium Raman activity at 1574 cm^−1^, in excellent agreement with the very strong and broad band observed in the IR spectrum and centered at 1577.8 cm^−1^, in which this mode contributes, in addition to the δ(O-H) of the hydrated water molecules. A similar result was obtained at the M06-2X/Lanl2dz level, but it was predicted at 1581 cm^−1^, with the highest IR intensity of the spectrum. In the 2b molecule alone [29], it was scaled at 1556 cm^−1^, in accordance with the experimental IR band with medium intensity at 1543.9 cm^−1^ and the Raman line at 1550.6 cm^−1^.

#### 2.4.3. The Aryl Ring Modes

The assignments for several aryl ring modes are obvious and require no further discussion; therefore, our attention was only focused on the strongest vibrations to confirm the structure of the synthesized complex. For the assignments of the ring modes, we followed the Varsanyi notation [42] for a 1,4-disubstituted benzene.

The aromatic C-H stretching vibrations are generally observed in the 3200–2950 cm^−1^ range, and they are predicted theoretically as almost pure modes (100% PED) with weak and very weak IR intensity and weak Raman activity. Therefore, the only **2b** mode with a scaled wavenumber at 2956 cm^−1^ was related to the experimental IR band at 2968.1 cm^−1^.

The aromatic C-C stretching vibration modes, modes **8a** and **8b**, are characterized as almost pure modes with %PED higher than 90%, and they are observed in the experimental spectra at similar wavenumbers. However, mode **8a** is predicted with almost null IR intensity and with the highest Raman intensity. This is in good agreement with the very strong Raman line observed at 1597 cm^−1^. Mode **8b** is predicted with almost null IR and Raman intensity, and thus, it was not observed in the experimental spectra.

Mode **19a** is predicted at the M06-2X/Lanl2mb level with strong IR and Raman intensity at 1511 cm^−1^, in good agreement with the strong experimental IR band at 1500.2 cm^−1^ and the Raman line 1504 cm^−1^. The C-C stretching mode, mode **14**, appears to be predicted at 1234 cm^−1^, with medium IR intensity and null Raman intensity, in accordance with the experimental IR band with medium intensity observed at 1218.0 cm^−1^ and with its non-detection in the Raman spectrum.

#### 2.4.4. Comparison with Other Lanthanoid Ln(2b′)_3_ Complexes

In the present study, with the La(2b′)_3_ complex, the IR spectrum recorded appears to be very close to that found with other lanthanoid ions [33,48], having important biological activities, as expected. Only a few small changes can be observed. This similarity was also observed in a comparison with the Raman spectra. Thus, the very broad IR band centered at ca. 3402 cm^−1^ found with the La(III) ion and corresponding to the ν(O-H) stretching mode shifts up to 3421 cm^−1^ with Dy(III) and Gd(III) ions due to the slight change in the arrangement of the hydration water molecules [33]. This arrangement also slightly affects the IR intensity of other broad bands, such as that at 1578 cm^−1^ and assigned to the stretching ν(C8-N14) + 8a, ν(CC) mode. Therefore, the assignment of the bands carried out for the 2b′ ligand can mostly be applied to the bands of other lanthanide Ln(2b′)_3_ complexes. This feature indicates that the geometric structure of the ligands is affected very little by the different Ln ions.

### 2.5. Photophysical Properties

With the aim of obtaining the appropriated photophysical properties of the lanthanum complex under study, the effect of the sample state in solid or solution phase on these properties, as well as the effect of the lanthanide ion in the complex, was analyzed.

#### 2.5.1. Photophysical Properties of the 2b′ Free Ligand and in Its La Complex

**Figure 11:**
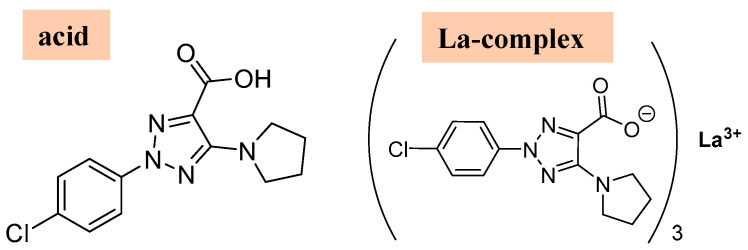
Molecular structures of the 2b′ ligand in its acid form and in its La(2b′)_3_ La-complex form, respectively.

The experimental absorbance and fluorescence photophysical properties of the 2b′ free ligand in its acid form (Figure 11) were compared to those determined in its La(2b′)_3_ complex. The samples were measured in DMSO solvent and in a mixture DMSO-H_2_O solution in a (1:9) proportion. The sample concentration was always 5.0 μM. The results obtained are included in Table 6.

**Figure 12:**
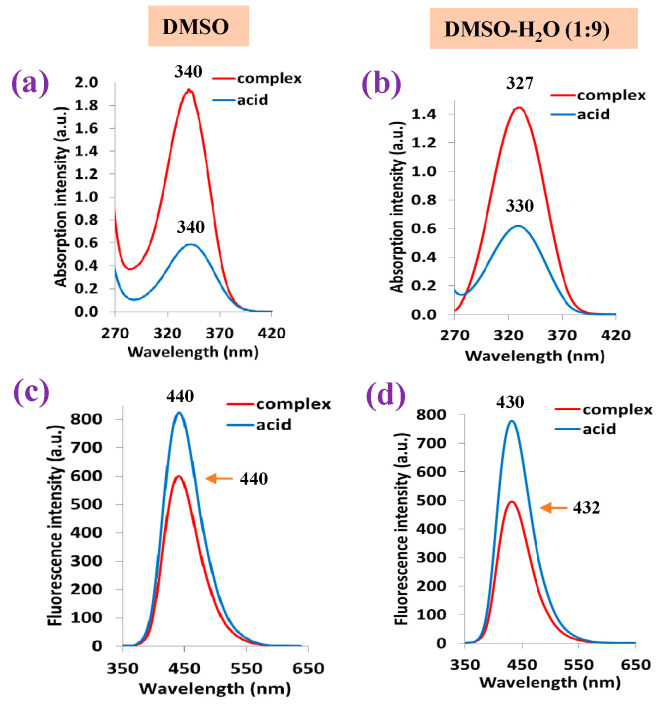
Absorption and fluorescence spectra of the 2b′ free ligand in its acid form and in its La complex: (**a**,**c**) results when using DMSO solution and (**b**,**d**) results when using DMSO-H_2_O (1:9) mixture solution.

A graphical comparison of the shape and wavelength position of the strongest absorption band obtained for the 2b′ free ligand in its acid form vs. that in its lanthanum La(2b′)_3_ complex in DMSO solution form is shown in Figure 12a. The absorbance position at 340 nm is not affected by the sample state in its free or complex form; however, the absorbance intensity is ca. four times higher in the complex than in the free form. When the sample is in a DMSO-H_2_O mixture (Figure 12b), the wavelength position shifts to a lower value. This shift is slightly larger in the complex than in the free form. Additionally, the absorption intensity is noticeably reduced in the complex form, while it remains almost unchanged in the free form. The high water polarity in the mixture solution appears to have a large effect in both the complex and free sample.

Similarly to that observed in the absorption spectrum, the fluorescence band position at 440 nm is not affected by the sample state, although in this case, the fluorescence intensity appears larger in the acid free form than in the complex form (Figure 12c). The DMSO-H_2_O mixture reduces the fluorescence intensity in both the acid-free form and complex form, while also shifting the band position to a slightly lower wavelength (Figure 12d).

#### 2.5.2. Photophysical Properties of Lanthanide Complexes

**Figure 13:**
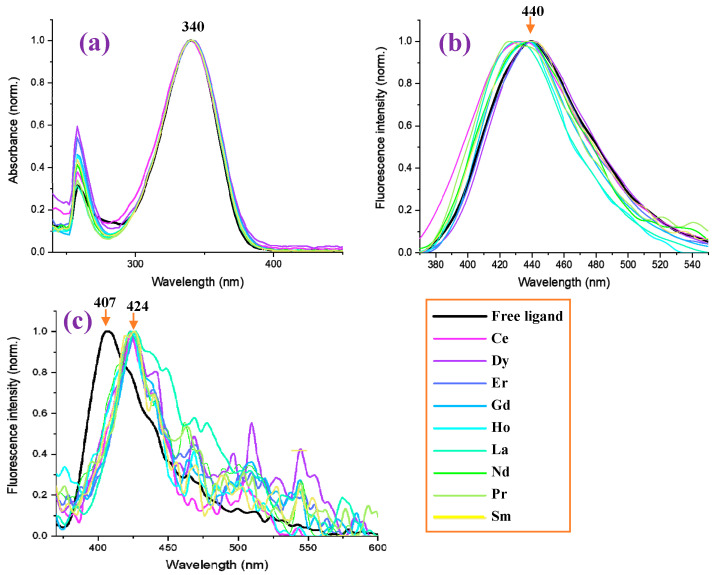
Measured photophysical properties of free ligand (2b′) and in nine of its lanthanide Ln(2b′)_3_ complexes. (**a**) Normalized absorbance of the samples in DMSO at a concentration of 1 × 10^−5^ M. (**b**) Normalized photoluminescence of the samples in DMSO at a concentration of 1 × 10^−5^ M. (**c**) Normalized photoluminescence of the samples in solid powder form.

The absorbance values measured in the 2b′ free ligand, as well as in their lanthanide complexes Ln(2b′)_3_ with Ln = La, Ce, Pr, Nd, Pr, Sm, Er, Gd, Ho, Er in DMSO solution, are shown in Figure 13a. It is observed that the graph with the absorbance of the free ligand in the anion form is close to that of all Ln complexes, having the same absorbance maximum at 340 nm. Similarly to that observed in the IR and Raman spectra, the Ln(III) ion type does not have a significant effect on the spectra absorbance. Moreover, the absorbance measured comes from the ligand, with the complex formation’s effect being almost null.

By contrast, the Ln(III) ion type has a significant effect for the fluorescence spectra in DMSO solution, and in some complexes, a blue shift in their values with respect to the 2b′ free ligand can be observed; see Figure 13b. The strongest blue shift (15 nm) can be observed for the Pr(2b′)_3_ complex with a maximum at 425 nm, while the maximum with the free ligand 2b′ appears at 440 nm. Similar shifts can be observed for the Ho(2b′)_3_ and Ce(2b′)_3_ complexes. In turn, the Dy(2b′)_3_ and Er(2b′)_3_ complexes show the same fluorescence spectra as the free ligand.

In the solid-state sample, the fluorescence spectrum of the 2b′ free ligand shows a maximum at 407 nm (Figure 13c), while a red shift of ca. 20 nm appears in their complexes with a maximum at 424 nm. The fluorescence maximum of all these complexes is similar, but the fluorescence intensity decreases with the wavelength in a different way with the Ln(2b′)_3_ complex; see Figure 13c. The fluorescence emission in their complexes is greatly diminished with lots of scattering due to their low emission.

**Figure 14:**
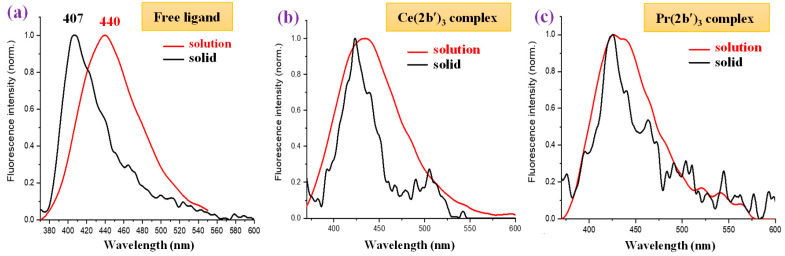
Comparison of the fluorescence emission of the samples in DMSO solution vs. power solid state of (**a**) free ligand (2b′), (**b**) Ce(2b′)_3_ complex, and (**c**) Pr(2b′)_3_ complex.

Comparing the fluorescence of all samples in the solid state with that in DMSO solution (Figure 14), it can be observed that a general blue shift in the powder solid sample with respect to solution occurs, with the highest blue shift being a shift of 33 nm in the 2b′ ligand (Figure 14a), probably due to the appearance of H-aggregates of it in the solid state. This blue shift in powder is small in all complexes, which may be due to the absence of these H-aggregates in the solid state. Figure 14b,c show a comparison of this only for the Ce(2b′)_3_ and Pr(2b′)_3_ complexes.

**Figure 15:**
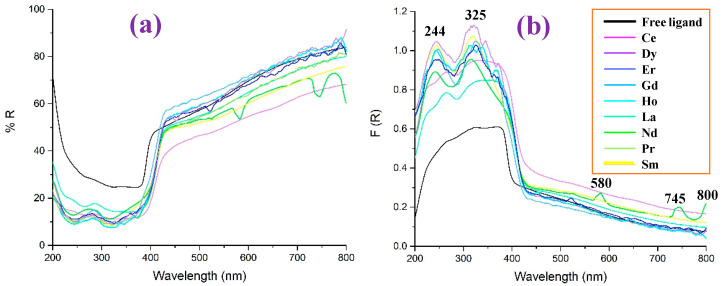
Comparison in the 2b′ free ligand and their lanthanide Ln(2b′)_3_ complexes of: (**a**) the reflectance UV-Vis values (% R) vs. the wavelength, (**b**) the diffuse reflectance F(R) vs. the wavelength.

The reflectance UV-Vis values (% R) in the 2b′ free ligand, as well as in their lanthanide complexes, Ln(2b′)_3_, are shown in Figure 15a as a function of the wavelength, while the diffuse reflectance F(R) in them is shown in Figure 15b. In general, all complexes show a similar reflectance pattern. However, the 2b′ free ligand shows remarkably higher values of R and lower values of F(R) in the 200–400 nm range and is without peaks in this range.

Comparing the diffuse reflectance spectra, it can be seen more clearly that the incorporation of the lanthanides produces two absorption peaks around 244 and 325 nm, the intensity of which is less significant for the La and Ce complexes, while Dy stands out. The behavior of Nd is also interesting, since it is the only ion in which three extra peaks appear at 580, 745, and 800 nm.

## 3. Materials and Methods

### 3.1. Experimental Details

The acid and complexes were synthesized according to previously reported procedures [49] via an interaction between lanthanum(III) inorganic salt and the ligand in an amount equal to a metal/ligand molar ratio of 1:3. The synthesis was carried out by adding an aqueous solution of La(III) nitrate to an aqueous solution of the ligand. The precipitate was filtered, washed with water, and dried in a desiccator until constant weight.

A solid-state infrared spectrum of the complex was recorded in KBr in the 4000–400 cm^−1^ frequency range using an FTIR IFS25 Bruker spectrometer (Bruker Company, Billerica, MA, USA). Raman spectra of the acidic form (**1b**) and the sodium salt (**2b**) of the ligand and its new La(III) complex were recorded with a Dilor microspectrometer (Horiba-Jobin-Yvon, model LabRam) (Horiba group, Kyoto, Japan) equipped with a 1800 grooves/mm holographic grating. The 514.5 nm line of an argon ion laser (Spectra Physics, model 2016) (Spectra-Physics company, Stahnsdorf, Germany) was used for probe excitation. The spectra were collected in backscattering geometry with a confocal Raman microscope equipped with an OlympusLMPlanFL 50× objective lens. Raman signal detection was carried out with a Peltier-cooled CCD camera. A laser power of 100 mW was used in our measurements.

The photophysical properties of lanthanide complexes were measured using the following equipment: The Jenway 7315 spectrophotometer (Jenway Company, Hong-Kong, China) was used for measuring the absorbance of all samples in DMSO solution at 1 × 10^−5^ M concentration. The Perkin Elmer FL 8500 spectrophotometer (Perkin-Elmer company, Massachusetts, USA) was used to determine the fluorescence of all samples in DMSO solution at the same 1 × 10^−5^ M concentration. The LAMBDA 850+ UV/vis Spectrophotometer (Perkin Elmer) equipped with an integrating sphere and polytetrafluoroethylene (PTFE) as reference was used to measure the absorbance of all samples in solid state.

The absorption spectra of the acid and its La complex in DMSO solution, as well as in a DMSO-H_2_O (1:9) mixture, were recorded using a Shimadzu UV-1800 spectrophotometer from Kyoto, Japan. The fluorescence of the sample solutions was measured with a Hitachi F-7000 spectrophotometer, also made by a company based in Tokyo, Japan. The absolute quantum yield was determined using a Horiba FlouroMax 4 Spectrofluorometer (Horiba group, Kyoto, Japan), along with a Quanta-φ integrating sphere and FluorEssence 3.5 software.

### 3.2. Computational Details

Methods based on Density Functional Theory (DFT) [50] were only used for the calculations due to the large size of the La(2b′)_3_ complex. DFT computations have also provided results in biomolecules which are quantitatively in good accordance with those obtained at the Hartree-Fock (HF)/Møller-Plesset (MP2) level [51,52,53,54,55] and much better for the vibrational wavenumber calculations. The Minnesota functional M06-2X [56,57,58] DFT method was selected because it is the best choice among other meta-generalized gradient functionals to examine dispersion-bound systems [59,60]. Since noncovalent weak interactions are expected in our large system, this was the preferred method. In addition, this method has also shown broad applicability in chemistry [57]. The B3LYP [61,62] DFT method was also used because it leads to excellent results in IR and Raman vibrational wavenumber calculations [40,41]. The CAM-B3LYP DFT method [63] was also chosen since it also provides good results for noncovalent weak interactions, which are expected in such large systems. All these methods were implemented in the GAUSSIAN-16 program package (Gaussian company, Wallingford, CT, USA) [64]. We ran the UNIX version with this package’s standard parameters in the Brigit super computer of the University Complutense at Madrid.

For lanthanide atoms, few basis sets appear available. The Lanl2dz [65] basis set was chosen because it gave good results in a La(III) complex with a carboxylic acid derivative [14]. Similarly, the Lanl2mb basis set [66] was also used. Another type available is the CEP-4g [67], but it is a very small basis set that can be only used for compounds with heavier metal atoms than lanthanum.

The global chemical reactivity descriptors were calculated using the following formulae:

IP = −E_HOMO_ (1)

EA = −E_LUMO_ (2)

χ = −(E_HOMO_ + E_LUMO_)/2 (3)

η = (E_LUMO_ − E_HOMO_)/2 (4)

S = ½ η(5)

Harmonic wavenumber calculations were carried out at the same level of the corresponding optimization process. All optimized structures show only positive harmonic vibrations, which indicate a local minima energy. The calculated Raman scattering activities (*S*_i_) were converted into relative Raman intensities (*I_i_*) using the following relationship derived from the basic theory of Raman scattering [68]:

(6) $$I_{i}=\;\frac{{f\;\left(\nu_{o}-\;\nu_{i}\right)}^{4\;\;}S_{i}}{\nu_{i}\left[1\;-e^{-\left(\frac{hc\nu_{i}}{kT}\right)}\right]}$$

The symbols in the above equation are defined as follows: ν*_o_*: the frequency (cm^−1^) of exciting radiation; *ν_i_*: the vibrational frequency of the ith normal mode; *h*: Planck constant, *c*; speed of light; *k*: Boltzmann constant; *T*: absolute temperature; and *f*: a suitably chosen scaling factor common to all the band intensities.

#### Scaling the Wavenumbers

To correct the overestimation in the frequency calculation by the theoretical methods used, it was necessary to use different scaling procedures specially selected for each level [40,41], therefore allowing for a remarkable improvement in the results. This is the regular procedure followed to obtain an accurate assignment of the experimental bands. The Linear Scaling Equation (LSE) procedure and the Polynomic Scaling Equation (PSE) procedure were mainly used, and they are as follows:

ν^scal^ = 16.6 + 0.9401·ν^cal^ at M06-2X/Lanl2dz level (LSE procedure) (7)

ν^scal^ = −22.0 + 0.9935·ν^cal^ − 1.33 × 10^−5^·(ν^cal^)^2^ at M06-2X/Lanl2dz level (PSE procedure) (8)

ν^scal^ = 67.6 + 0.8502·ν^cal^ at M06-2X/Lanl2mb level (LSE procedure) (9)

ν^scal^ = −13.1 + 0.9555·ν^cal^ − 2.43 × 10^−5^ ·(ν^cal^)^2^ at M06-2X/Lanl2mb level (PSE procedure) (10)

ν^scal^ = 6.4 + 0.9463·ν^cal^ at CAM-B3LYP/Lanl2dz level (LSE procedure) (11)

ν^scal^ = −26.3 + 0.9914·ν^cal^ − 1.13 × 10^−5^·(ν^cal^)^2^ at CAM-B3LYP/Lanl2dz level (PSE) (12)

ν^scal^ = 62.5 + 0.8576·ν^cal^ at CAM-B3LYP/Lanl2mb level (LSE procedure) (13)

ν^scal^ = −12.6 + 0.9560·ν^cal^ − 2.28 × 10^−5^·(ν^cal^)^2^ at CAM-B3LYP/Lanl2mb level (PSE) (14)

## 4. Summary and Conclusions

A detailed study of the experimental IR and Raman spectra of a hydrated La(2b′)_3_ cluster was carried out, and its structure and spectroscopy were well characterized with the help of four DFT levels. The most important findings are as follows:

- The optimized La(III) complex at the CAM-B3LYP/Lanl2dz and M06-2X/Lanl2dz levels show a symmetrical arrangement of the ligands, while at B3LYP/Cep-4g and especially at M06-2X/Lanl2mb, a deformed structure with packing interaction of two ligands was obtained.
- The hydration water molecules present in the experimental sample were considered under the DM model with different numbers of explicit water molecules around the La(III) ion and the carboxylate groups and in different positions. The theoretical spectra obtained at different DFT levels were compared to the experimental one. The optimized cluster at the M06-2X/Lanl2dz level appears less deformed and has an IR spectrum better resembling the experimental one, compared to those at the CAM-B3LYP/Lanl2dz and M06-2X/Lanl2mb levels.
- By comparing the scaled spectrum at the M06-2X/Lanl2dz level with the experimental broad band intensity at 3401.6 cm^−1^, the number of hydration water molecules was predicted to be seven or eight. The distribution of these water molecules appears to be around the active centers of the complex, not only the La(III) ion or the carboxylate groups, but also the nitrogen atoms. These H-bonds/interactions should not be very strong because, in the experimental spectrum, bands corresponding to strongly H-bonded water molecules do not appear.
- Several new scaling equations were used to improve the calculated spectra. In the isolated state, the scaled spectra at the M06-2X/Lanl2mb level appear closest to the experimental one due to its packing form. However, this level dramatically fails in the calculation of the hydrated form spectrum. With the scaling, the worst fit with experimental data was observed at the B3LYP/Cep-4g level, and therefore, the results achieved with this level were not considered.
- The scaled wavenumbers of the most intense IR and Raman vibrations in the spectrum at M06-2X/Lanl2dz level appear to show the best adherence, based on both frequency and intensity, to the most intense experimental IR and Raman bands. Therefore, the spatial arrangement of the ligands in the synthesized La(III) complex was confirmed, as well as the assignments of the bands.
- The O-H bending of the water molecules contributes to the broadening of the experimental IR band at 1577.8 cm^−1^.
- The HOMO orbital shows the charge density localized over the triazole ring and the neighboring carbon atoms, while in the LUMO orbital, the charge distribution appears mainly on the oxygen and lanthanum ion. The hydration water affects the orbital energies very little, and only a slight reduction in negative HOMO energy was observed.
- The calculated molecular properties and global chemical descriptors of this complex indicate large chemical reactivity and low excitation energies for the excited states.
- Large similarity was observed in the comparison of the IR and Raman spectra of La(2b′)_3_ with that obtained with other lanthanide ions. Only small shifts appear in the very broad IR band corresponding to the ν(O-H) stretching mode, due to the different arrangements of the hydration water molecules.
- Photophysical properties were also investigated. The absorbance of the free ligand in DMSO solution is similar to that obtained in all Ln complexes, with the same absorbance maximum at 340 nm. However, a blue shift in the fluorescence spectra in DMSO solution is observed in the complexes compared to the free ligand. In addition, a general blue shift in the fluorescence is observed in the solid-state sample with respect to that in solution, probably due to the appearance of H-aggregates.
- Lanthanide complexes exhibit two distinct absorption peaks in the diffuse reflectance F(R) spectra, while the free ligand shows remarkably higher reflectance values, UV-Vis values, and a lower F(R) value in the 200–400 nm range.

These spectroscopic findings explain the molecular structure of a lanthanum complex, highlighting its considerable chemical reactivity and its potential application as an antioxidant, antimicrobial, or anticancer agent.

## Figures and Tables

**Table 1:** Several selected optimized geometrical parameters calculated at different DFT levels in ligand *III* of the La(2b’)_3_ complex. Bond lengths (r) are in Å; bond angles and dihedral angles (∠) are in degrees. The symbol (′) on an atom indicates that it corresponds to ligand *I*, and the symbol (′′) to ligand *II.*

Parameters	2b’	La(2b’)_3_ Isolated State				La(2b’)_3_ + 10 H_2_O	
	MP2	B3LYP	CAM-B3LYP	M06-2X		CAM-B3LYP	M06-2X
	6-31G(d,p)	Cep-4g	Lanl2dz	Lanl2dz	Lanl2mb	Lanl2dz	Lanl2dz
r(C_4_-N_4_)	1.397	1.538	1.422	1.421	1.450	1.422	1.418
r(N_4_-N_7_)	1.350	1.510	1.373	1.375	1.420	1.366	1.374
r(N_4_-N_10_)	1.352	1.480	1.330	1.330	1.391	1.342	1.341
r(C_8_-C_9_)	1.430	1.566	1.448	1.447	1.446	1.437	1.444
r(C_9_-N_10_)	1.348	1.487	1.358	1.359	1.372	1.350	1.350
r(C_9_-C_11_)	1.541	1.579	1.456	1.455	1.507	1.482	1.472
r(C=O_12_)	1.267	1.418	1.310	1.310	1.307	1.303	1.308
r(C=O_13_)	1.252	1.428	1.306	1.305	1.325	1.276	1.284
r(La-O_12_)	-	2.257	2.525	2.523	2.460	2.480	2.571
r(La-O_13_)	-	3.646	2.486	2.491	2.333	3.900	2.767
O_12_···H_18_	2.034	2.257	2.464	2.443	1.867	2.323	2.139
∠(C_4_-N_4_-N_7_)	121.9	120.7	122.0	121.9	121.6	122.5	122.2
∠(C_4_-N_4_-N_10_)	122.0	119.8	122.8	122.8	121.7	122.8	122.9
∠(N-N-N)	116.1	119.4	115.2	115.4	116.7	114.7	114.9
∠(N_10_-C_9_-C_11_)	120.7	119.9	119.1	119.5	117.6	120.8	120.9
∠(N_7_-C_8_-N_14_)	119.2	120.8	119.5	119.8	120.8	120.9	121.2
∠(C_9_-C_8_-N_14_)	130.9	129.5	133.0	132.6	128.7	130.8	131.0
∠(C_9_-C_11_=O_12_)	113.3	121.3	121.7	121.2	123.3	117.3	117.5
∠(C_9_-C_11_=O_13_)	115.4	120.7	121.2	121.4	118.8	119.5	122.5
∠(O=C=O)	131.3	117.9	117.1	117.4	117.8	123.1	120.0
∠(C=O_12_-La)	-	87.9	94.0	94.0	87.1	137.3	98.6
∠(C=O_13_-La)	-	89.0	95.9	95.7	92.2	-	90.2
∠(O_12_-La-O′_13_)	-	95.2	132.0	132.6	79.8	120.6	116.7
∠(O_13_-La-O′_12_)	-	119.2	121.9	121.8	132.8	-	115.4
∠(C_5_-C_4_-N_4_-N_10_)	−2.3	−3.5	1.1	0.8	1.5	−1.1	−3.1
∠(C_4_-N_4_-N_10_-C_9_)	−178.8	−177.9	179.0	178.9	−178.6	−179.1	−178.1
∠(N_4_-N_10_-C_9_-C_11_)	175.9	177.7	−175.3	−174.6	−176.9	173.6	171.7
∠(C_8_-N_7_-N_4_-N_10_)	−1.4	−0.6	0.3	0.3	0.7	−0.3	−0.6
∠(N_10_-C_9_-C=O_12_)	−145.6	−149.9	167.0	166.8	160.0	−174.4	−151.5
∠(N_10_-C_9_-C-O_13_)	33.4	34.0	−11.2	−11.2	−16.5	2.8	26.1
∠(C_8_-C_9_-C=O_12_)	29.8	28.9	−8.1	−7.5	−16.7	−1.6	19.8
∠(C_11_-C_9_-C_8_-N_14_)	3.1	5.0	−5.8	−6.7	-0.8	5.3	9.1
∠(C_9_-C_8_-N_14_-C_15_)	172.2	167.6	176.3	176.8	−174.7	−172.2	−172.5
∠(C_9_-C_8_-N_14_-C_18_)	32.0	18.7	−16.1	−18.3	−31.2	53.7	35.3
∠(C_8_-N_14_-C_15_-C_16_)	−162.4	−140.6	−177.2	−179.4	159.9	−170.9	−166.0
∠(N_14_-C_15_-C_16_-C_17_)	−9.3	−29.4	−31.4	−32.2	11.6	40.3	−14.4
∠(C_11_-O_12_···O′_12_-C′_11_)	-	153.5	11.7	13.2	−169.5	90.5	3.4
∠(C_9_-C_11_···C′_11_-C′_9_)	-	−52.7	0.6	0.3	8.1	48.2	24.5
∠(C′_9_-C′_11_···C″_11_-C″_9_)	-	−21.1	−4.7	−3.2	6.8	−74.9	−66.7
∠(C_11_···La···C′_11_)	-	145.6	120.6	121.1	132.2	96.5	114.4
∠(C′_11_···La···C″_11_)		91.1	119.4	118.3	84.7	94.5	82.7

**Table 2:** APT (Atomic Polar Tensor) charges calculated at different DFT levels in La(2b’)_3_ complex. Only the values of ligand *I* are shown.

	2b′	La(2b′)_3_				La(2b′)_3_ + 10 H_2_O	
	MP2 *	B3LYP	CAM-B3LYP	M06-2X		CAM-B3LYP	M06-2X
Atom	6-31G(d,p)	Cep-4g	Lanl2dz	Lanl2dz	Lanl2mb	Lanl2dz	Lanl2dz
La	-	2.884	3.095	3.164	2.066	2.979	2.869
CL	−0.054	−0.254	−0.414	−0.423	−0.496	−0.411	−0.429
C_1_	−0.080	0.267	0.455	0.452	0.476	0.453	0.433
C_4_	0.210	0.620	0.528	0.518	0.723	0.548	0.618
N_4_	−0.073	−0.742	0.548	−0.566	−0.832	−0.529	−0.660
N_7_	−0.402	−0.066	−0.296	−0.293	−0.069	−0.192	−0.242
C_8_	0.419	0.373	0.713	0.714	0.465	0.544	0.623
C_9_	0.031	−0.844	−0.812	−0.835	−0.716	−0.503	−0.614
N_10_	−0.240	0.401	0.319	0.340	0.398	0.188	0.300
C_11_	0.955	1.381	1.738	1.821	1.506	1.576	1.635
O_12_	−0.887	−1.014	−1.195	−1.250	−0.925	−1.321	−1.262
O_13_	−0.839	−1.089	−1.214	−1.266	−1.033	−1.042	−1.078
N_14_	−0.557	−0.713	−0.907	−0.924	−0.771	−0.974	−0.922

* calculated in isolated 2b′ molecule [29].

**Table 3:** Molecular properties and global chemical descriptors (eV) calculated at three DFT levels in the La(2b**′**)_3_ complex.

	La(2b′)_3_				La(2b′)_3_ + 10 H_2_O	
Molecular Properties	B3LYP	CAM-B3LYP	M06-2X		CAM-B3LYP	M06-2X
	Cep-4g	Lanl2dz	Lanl2dz	Lanl2mb	Lanl2dz	Lanl2dz
Rotational constants (GHz):						
A	0.035	0.019	0.019	0.040	0.021	0.023
B	0.013	0.017	0.017	0.015	0.017	0.016
C	0.012	0.012	0.012	0.014	0.012	0.012
C_v_ (cal/mol·K)	229.86	202.7	202.97	200.6	279.5	284.6
S (cal/mol·K)	376.53	349.2	348.72	345.3	424.3	428.0
Dipole moment (Debye)	4.377	4.099	4.085	3.323	5.807	5.526
HOMO	−0.253	−0.263	−0.262	−0.189	−0.251	−0.251
LUMO	−0.146	−0.039	−0.052	0.009	−0.033	−0.052
E_g_	0.107	0.224	0.210	0.198	0.218	0.199
IP	0.253	0.263	0.262	0.189	0.251	0.251
EA	0.146	0.039	0.052	−0.009	0.033	0.052
χ	0.199	0.151	0.157	0.090	0.142	0.151
η	0.054	0.112	0.105	0.099	0.109	0.099
S	0.027	0.056	0.052	0.049	0.054	0.050

**Table 5:** Comparison of several main scaled and experimental wavenumbers (ν, cm^−1^) in the isolated state and in the hydration La(2b′)_3_ + 10 H_2_O cluster.

Isolated State		+10 H2O		Experimental	Characterization
CAM-B3LYP	M06-2X	CAM-B3LYP	M06-2X	IR	
1588 1474 1361 1266 1178 857 629 555 507	1581 1470 1361 1267 1184 814 668 599 520	1598 1486 1358 1275 1175 876 669 578 515	1602 1486 1365 1271 1181 854 639 559 508	1577.8 vs 1484.5 vs 1372.3 vs 1285.5 s 1178.2 m 829.7 vs 647.1 m 576.7 w 508.9 m	ν(C8-N14) + 8a, ν(CC) 19a, ν(CC) νs(COO) + νs(NNN) δas(C-H) in pyrrolidine ν(triazol) δas(COO) Γ(triazol) δ(NNN) δas(COO) in phase

**Table 6:** Photophysical data for the 2b′ ligand in its acid form and in its La complex form in DMSO solution and in DMSO-H_2_O (1:9) mixture solution for a concentration of 5.0 μM. Notations used: λ, wavelength of the absorption/emission band; ε, molar extinction coefficient.

		Absorbance		Fluorescence		
Solvent	Compound	λ_*max*_, nm	ε, M^−1^ cm^−1^	λ_*em*_, nm	Φ_*F*_, %	Stokes Shift, nm/cm^−1^
DMSO	Acid	340	12,000	440	71.1	100/6684
	La complex	340	32,200	440	41.0	100/6684
DMSO-H2O (1:9)	Acid	330	13,200	430	44.2	100/6684
	La complex	327	28,000	432	33.9	105/7433

## Data Availability

Data are contained within the article.

## Supplementary Materials

The following supporting information has been submitted in addition to the main body at: https://www.mdpi.com/article/10.3390/molecules30163412/s1, Figure S1: Front and lateral views of the optimized La(2b′)3 complex at the: (a) B3LYP/Cep-4g level, (b) M06-2X/Lanl2mb level; Figure S2: Optimized La(2b′)_3_ + 10 H_2_O cluster at the M06-2X/Lanl2mb level. (a) lateral view of the cluster. (b) An amplified view indicating in green circles: ① the carboxylic oxygen is not bonded to La(III) ion, ② a water proton is bonded to a carboxylic oxygen, ③ the water oxygen that has lost a proton; Figure S3: Optimized La(2b′)_3_ + 10 H_2_O cluster at the CAM-B3LYP/Lanl2dz level; Figure S4: Comparison of the theoretical scaled Raman spectra with the experimental ones; Figure S5: Comparison of the scaled Raman spectra in the 3400–2600 cm^−1^ range by three DFT methods; Figure S6: Comparison of the scaled Raman spectra in the 1800–1000 cm^−1^ range at B3LYP/Cep-4g; Figure S7: Comparison of the scaled Raman spectra in the 1000–50 cm^−1^ range by three DFT methods; Figure S8: Comparison of the theoretical scaled IR spectra with the experimental ones in the 3750–400 cm^−1^ range; Figure S9: Comparison of the theoretical scaled IR spectra at the M06-2X/Lanl2dz level with the experimental ones in the 3750–2600 cm^−1^ range. (a) Scaled spectrum in the isolated La(2b′)_3_ complex. (b) Scaled spectrum in the La(2b′)_3_ + 10 H_2_O cluster but with the water molecule bands subtracted from the spectrum. The symbol (#) on H_2_O represents the removing of its bands. (c) Scaled spectrum in the La(2b′)_3_ + 10 H_2_O cluster with all bands. (d) Experimental IR spectrum in the solid state sample; Figure S10: Comparison of the theoretical scaled IR at the M06-2X/Lanl2dz spectra with the experimental ones in the 1800–1000 cm^−1^ range. (a) Scaled spectrum in the isolated La(2b′)_3_ complex. (b) Scaled spectrum in the La(2b′)_3_ + 10 H_2_O cluster but with the water molecule bands subtracted from the spectrum. The symbol (#) on H_2_O represents the removing of its bands. (c) Scaled spectrum in the La(2b′)_3_ + 10 H_2_O cluster with all bands. (d) Experimental IR spectrum in the solid state sample; Figure S11: Comparison of the theoretical scaled IR at the M06-2X/Lanl2dz spectra with the experimental ones in the 1000–400 cm^−1^ range. (a) Scaled spectrum in the isolated La(2b′)_3_ complex. (b) Scaled spectrum in the La(2b′)_3_ + 10 H_2_O cluster but with the water molecule bands subtracted from the spectrum. (c) Scaled spectrum in the La(2b′)_3_ + 10 H_2_O cluster with all bands. (d) Experimental IR spectrum in the solid state sample; Table S1: Calculated, scaled and experimental wavenumbers (ν, cm^−1^) in the La(2b)_3_ complex. Relative infrared intensity (A) in %, relative Raman intensity (S) in %, and Raman depolarization ratios for plane (DP) and unpolarized incident light (DU). For each vibration of the tetramer, the wavenumber with the highest IR intensity is indicated in bold type and that with the highest Raman intensity is indicated in italic type. The relative IR and Raman intensities were only shown for these wavenumbers. DP and DU values were from most intense Raman line. The number of the ring mode corresponds to Wilson’s notation [42]; Table S2: Calculated, scaled and vibrational assignment at the M06-2X/Lanl2mb level, together with the experimental wavenumbers (ν, cm^−1^) in the La(2b)_3_ complex. Relative infrared intensity (A) in %, relative Raman intensity (S) in %, and Raman depolarization ratios for plane (DP) and unpolarized incident light (DU). For each vibration of the tetramer, the wavenumber with the highest IR intensity is indicated in bold type and that with the highest Raman intensity is indicated in italic type. The relative IR and Raman intensities were only shown for these wavenumbers. DP and DU values were from most intense Raman line. The number of the ring mode corresponds to Wilson’s notation [42]; Table S3: Calculated, scaled and vibrational assignment at the B3LYP/Cep-4g level, together the experimental wavenumbers (ν, cm^−1^) in the La(2b)_3_ complex. Relative infrared intensity (A) in %, relative Raman intensity (S) in %, and Raman depolarization ratios for plane (DP) and unpolarized incident light (DU). For each vibration of the tetramer, the wavenumber with the highest IR intensity is indicated in bold type and that with the highest Raman intensity is indicated in italic type. The relative IR and Raman intensities were only shown for these wavenumbers. DP and DU values were from most intense Raman line. The number of the ring mode corresponds to Wilson’s notation [42].
