# Supplementary material for: The Spectroscopic Characterization and Photophysical Properties of a Hydrated Lanthanum Ion Complex with a Triazole Ligand by Several DFT Methods
Source: Molecules. 2025 Aug 18;30(16):3412. doi: 10.3390/molecules30163412 (PMC12388395; doi:10.3390/molecules30163412)
Supplement: Supplementary file 1 [file molecules-30-03412-s001.zip › molecules-3748532-supplementary.pdf]

SUPPLEMENTARY MATERIALS

The Spectroscopic Characterization and Photophysical Proper-ties of a Hydrated Lanthanum ion Complex with a Triazole Ligand by Several DFT Methods

M. Alcolea Palafox [1]*, Lozan T. Todorov [2], Nataliya P. Belskaya [3], Javier Álvarez-Conde [4], Diana Díaz-García [4], Santiago Gómez-Ruiz [4], and Irena P. Kostova [2]

[1] Departamento de Química Física, Facultad de Ciencias Químicas, Universidad Complutense, Madrid-28040, Spain (alcolea@ucm.es)

[2] Department of Chemistry, Faculty of Pharmacy, Medical University – Sofia, 2 Dunav Str., Sofia, Bulgaria (ltodorov@pharmfac.mu-sofia.bg)

[3] Department of Technology for Organic Synthesis, Ural Federal University, 19 Mira Str., Yekaterinburg 620012, Russia; n.p.belskaya@urfu.ru

[4] COMET-NANO Group, Department of Biology and Geology, Physics and Inorganic Chemistry, E.S.C.E.T., Universidad Rey Juan Carlos, calle Tulipán s/n, E-28933, Móstoles (Madrid), Spain (javier.alvarez@urjc.es; diana.diaz@urjc.es; santiago.gomez@urjc.es)

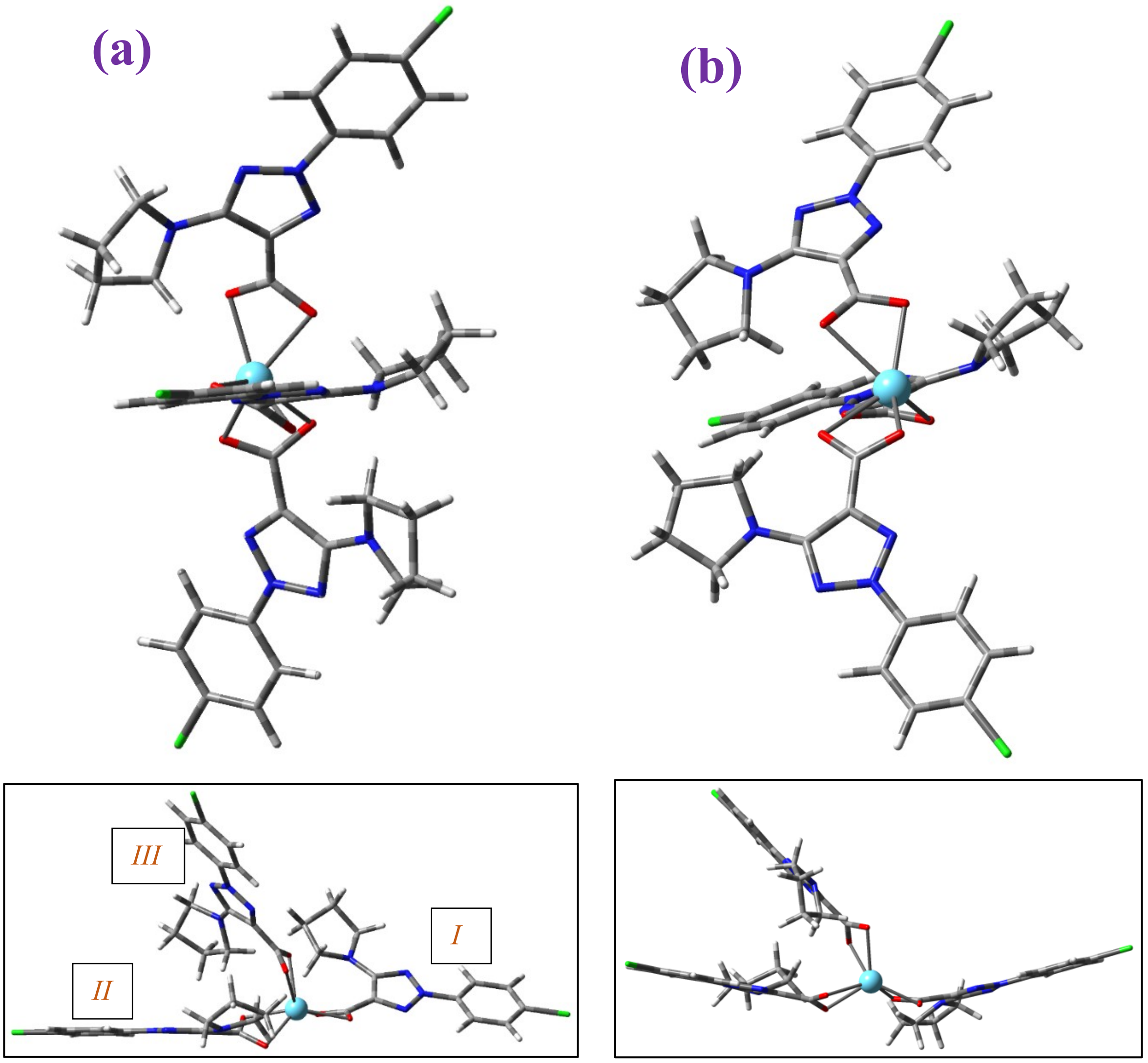

E = -528.387053 AU (*G* = -528.502017 AU)   *E* = -2658.358912 AU (*G* = -2658.466297 AU)

Figure S1. Front and lateral views of the optimized La(2b´)$_3$ complex at the: (a) B3LYP/Cep-4g level, (b) M06-2X/Lanl2mb level.

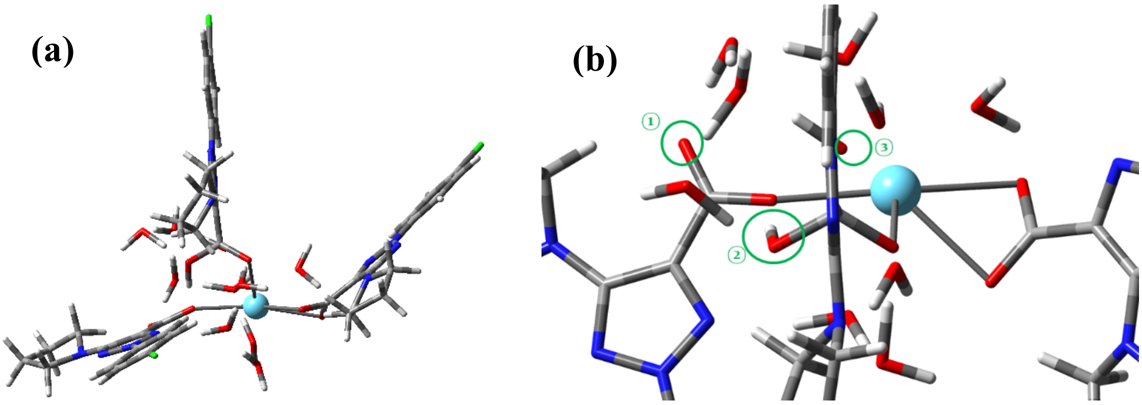

Figure S2. Optimized La(2b′)$_3$ + 10 H_2O cluster at the M06-2X/Lanl2mb level. (a) lateral view of the cluster. (b) An amplified view indicating in green circles: ① the carboxylic oxygen is not bonded to La(III) ion, ② a water proton is bonded to a carboxylic oxygen, ③ the water oxygen that has lost a proton.

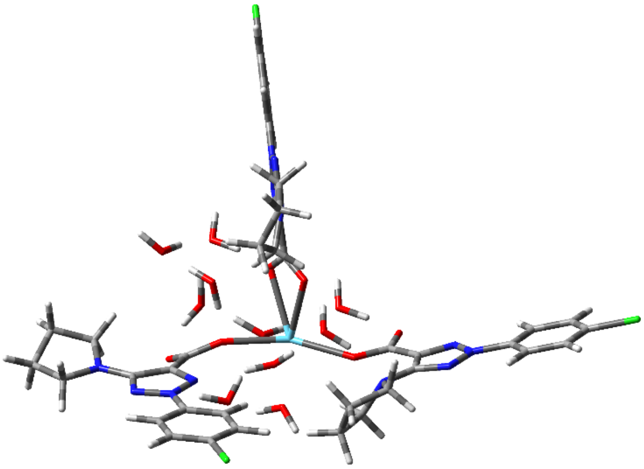

Figure S3. Optimized La(2b′)$_3$ + 10 H_2O cluster at the CAM-B3LYP/Lanl2dz level.

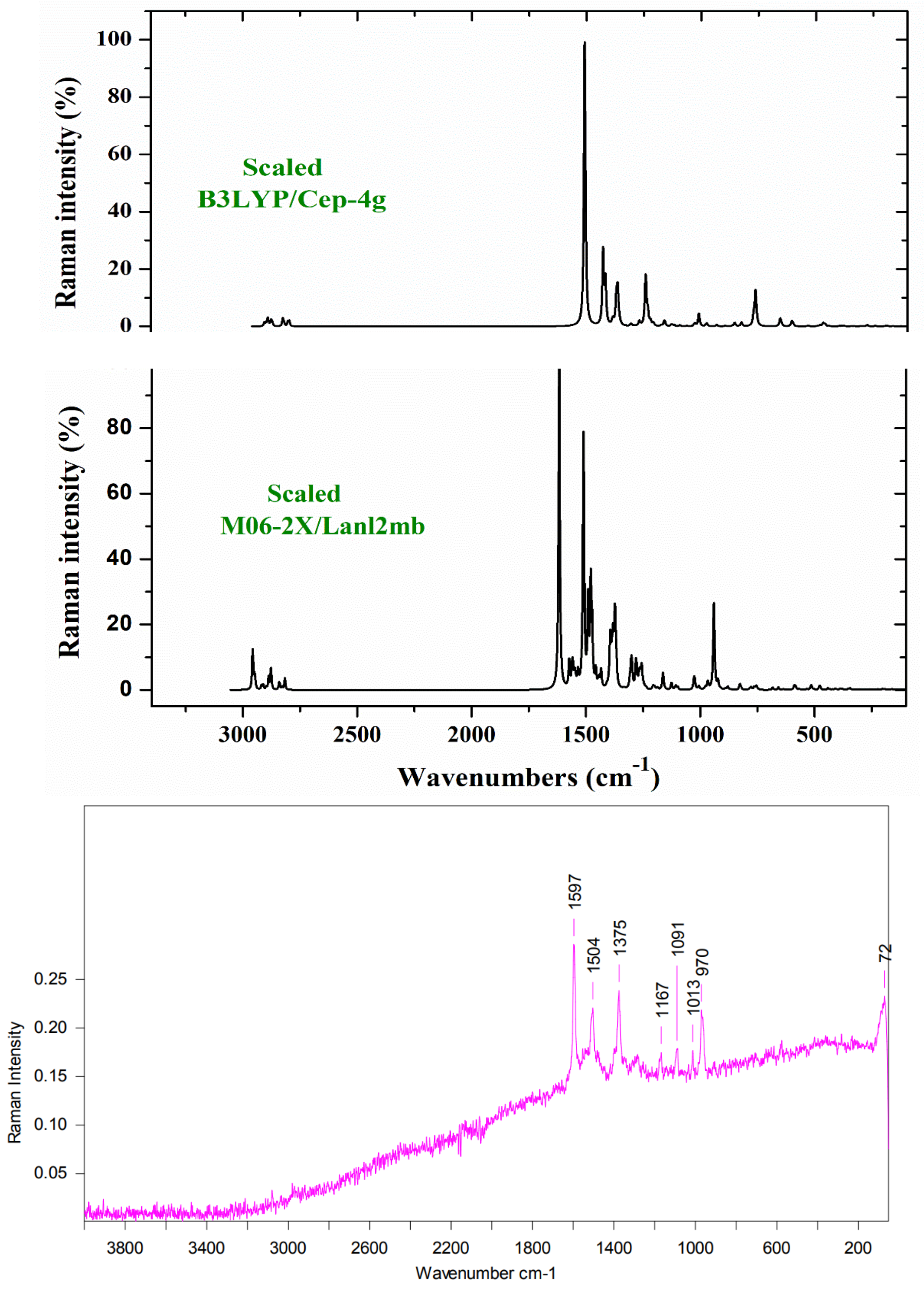

Figure S4. Comparison of the theoretical scaled Raman spectra with the experimental ones

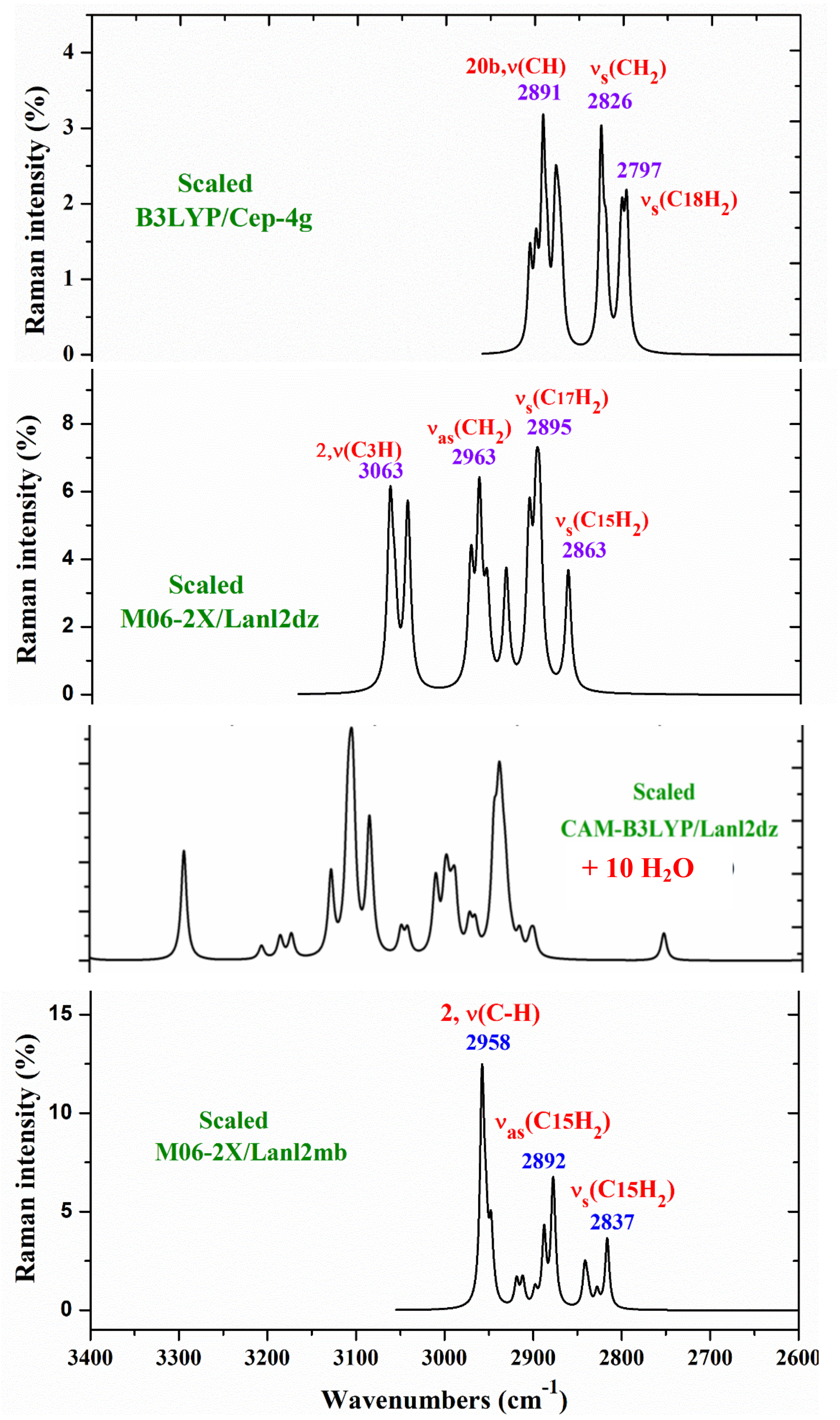

Figure S5. Comparison of the scaled Raman spectra in the 3400-2600 cm^{-1} range by three DFT methods.

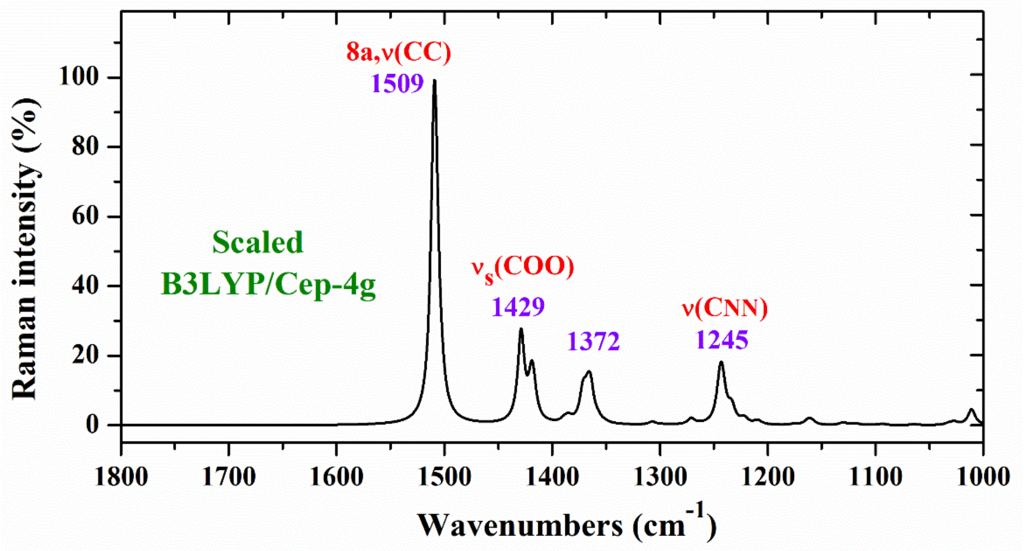

Figure S6. Comparison of the scaled Raman spectra in the 1800-1000 cm^{-1} range at B3LYP/Cep-4g.

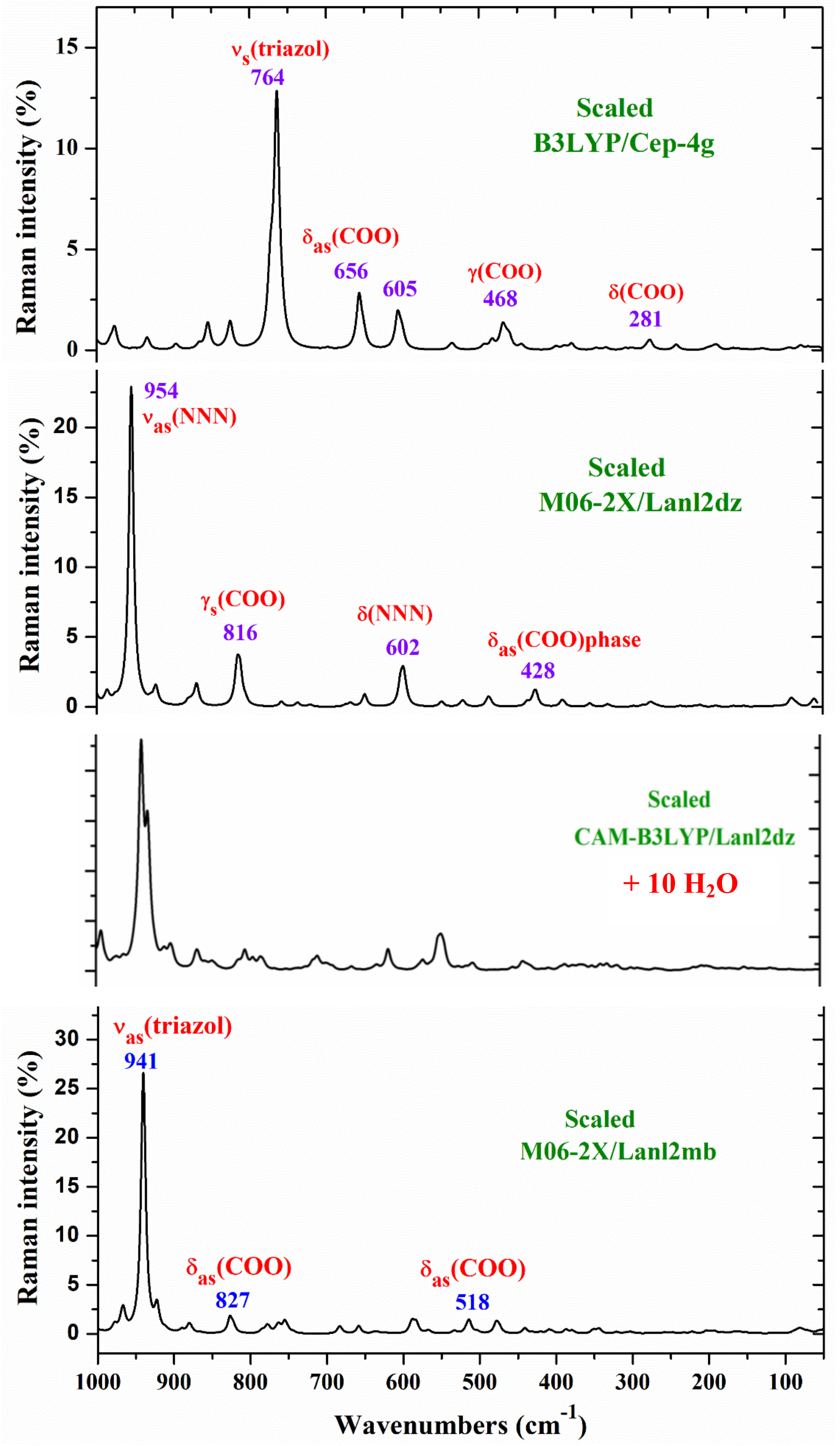

Figure S7. Comparison of the scaled Raman spectra in the 1000-50 cm^{-1} range by three DFT methods.

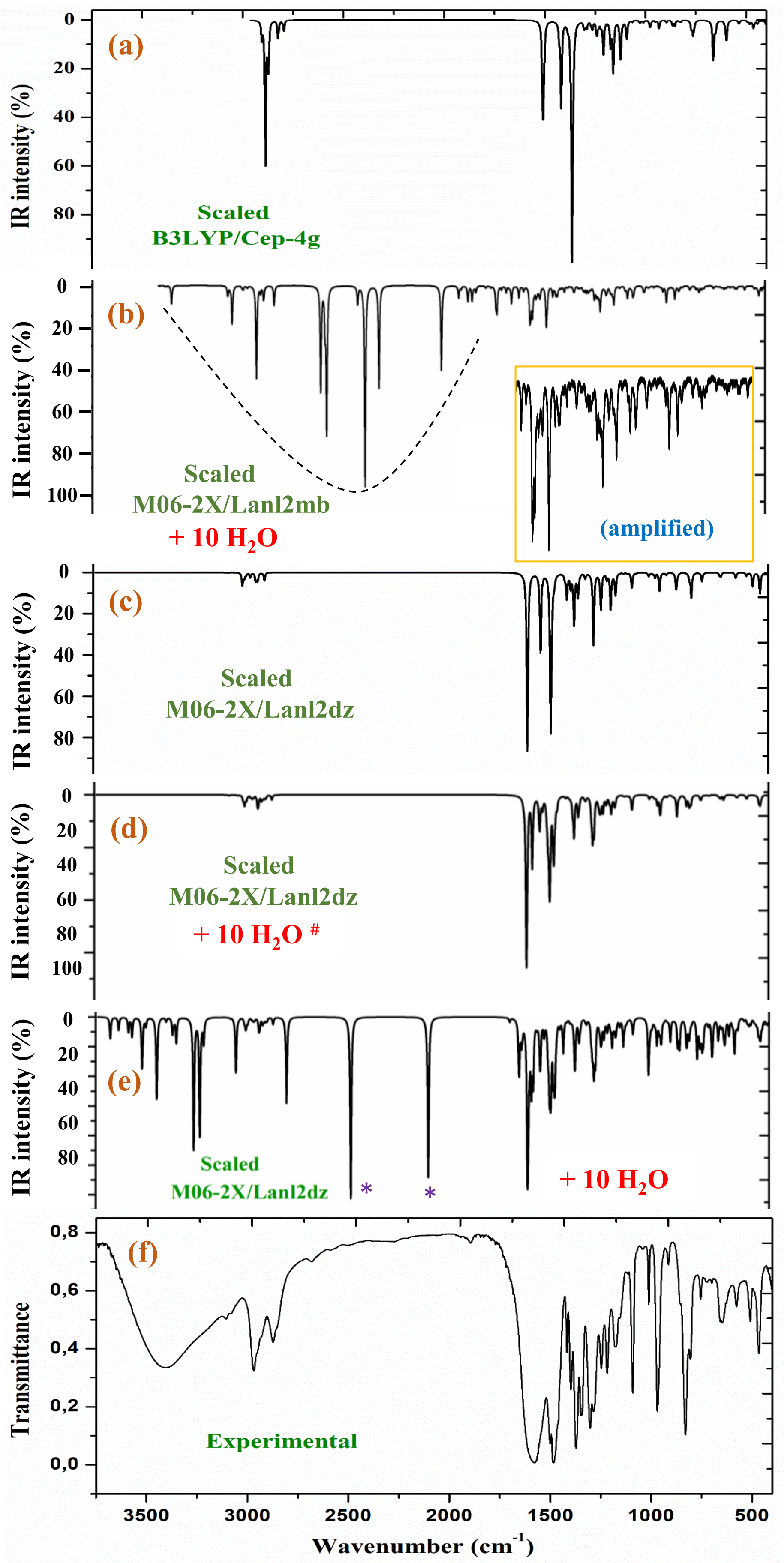

Figure S8. Comparison of the theoretical scaled IR spectra with the experimental ones in the 3750-400 cm^{-1} range.

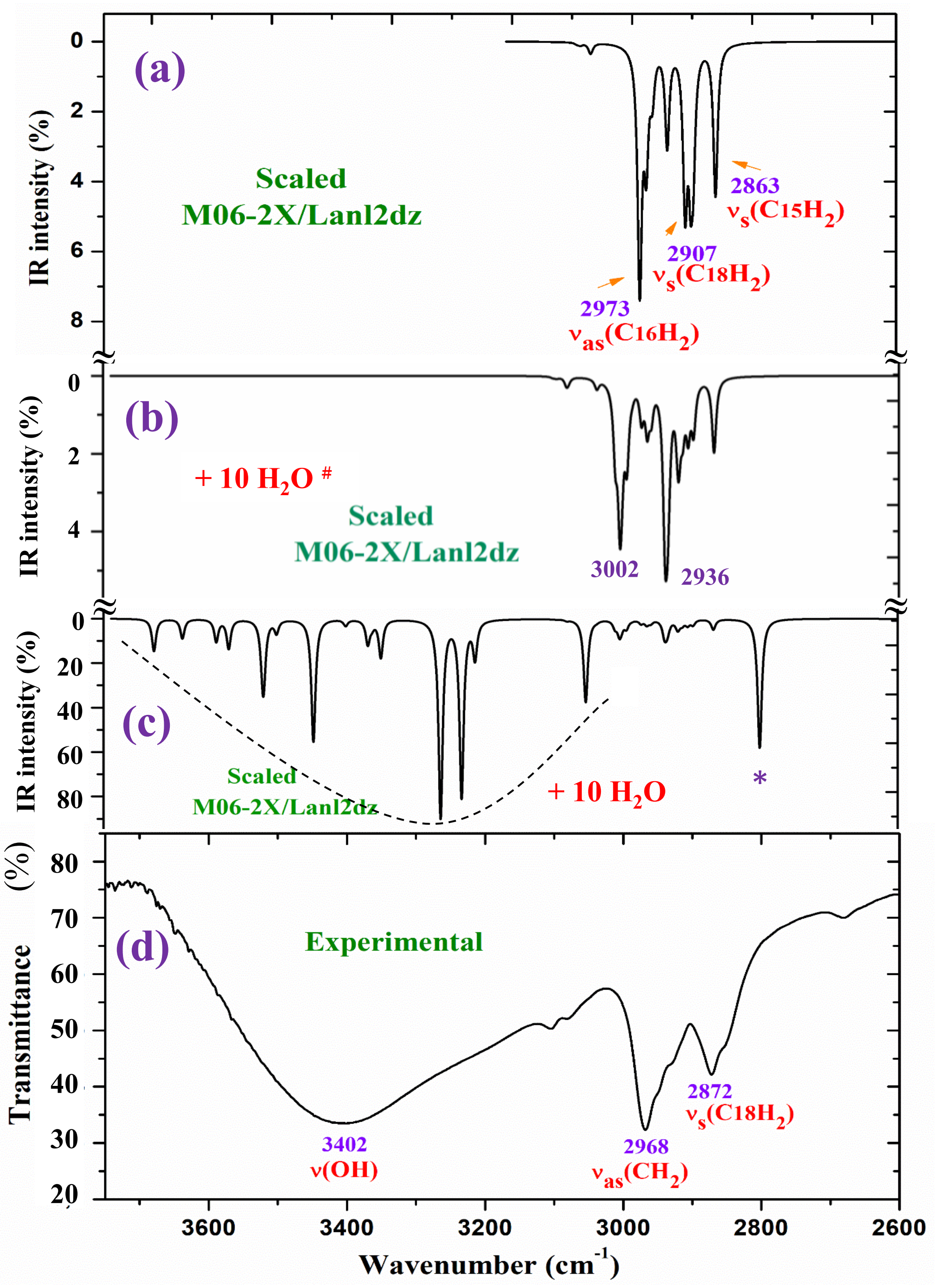

Figure S9. Comparison of the theoretical scaled IR spectra at the M06-2X/Lanl2dz level with the experimental ones in the 3750-2600 cm^{-1} range. (a) Scaled spectrum in the isolated La(2b′)$_3$ complex. (b) Scaled spectrum in the La(2b′)$_3$ + 10 H_2O cluster but with the water molecule bands subtracted from the spectrum. The symbol (#) on H_2O represents the removing of its bands. (c) Scaled spectrum in the La(2b′)$_3$ + 10 H_2O cluster with all bands. (d) Experimental IR spectrum in the solid state sample.

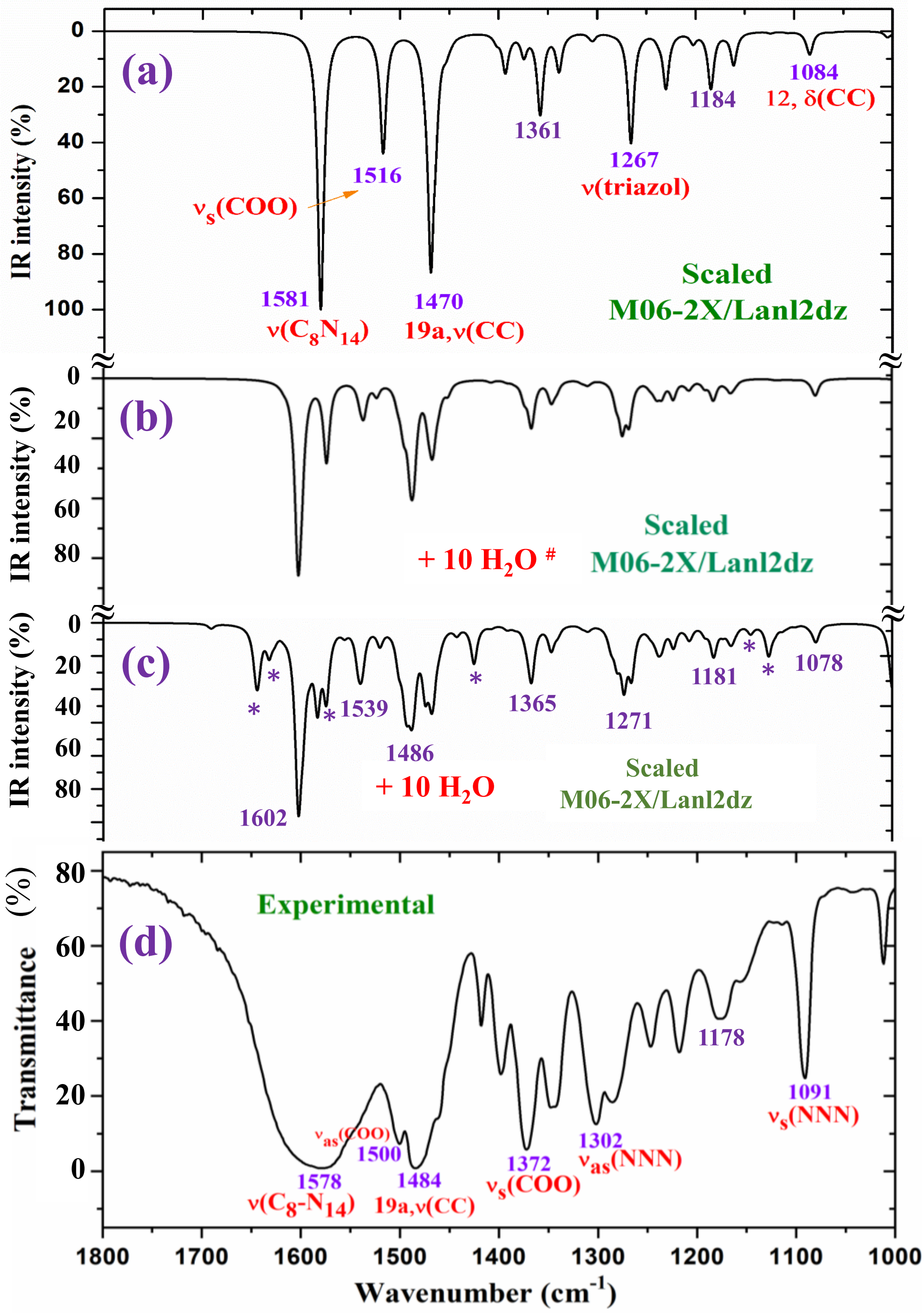

Figure S10. Comparison of the theoretical scaled IR at the M06-2X/Lanl2dz spectra with the experimental ones in the 1800-1000 cm^{-1} range. (a) Scaled spectrum in the isolated La(2b')$_3$ complex. (b) Scaled spectrum in the La(2b′)$_3$ + 10 H_2O cluster but with the water molecule bands subtracted from the spectrum. The symbol (#) on H_2O represents the removing of its bands. (c) Scaled spectrum in the La(2b′)$_3$ + 10 H_2O cluster with all bands. (d) Experimental IR spectrum in the solid state sample.

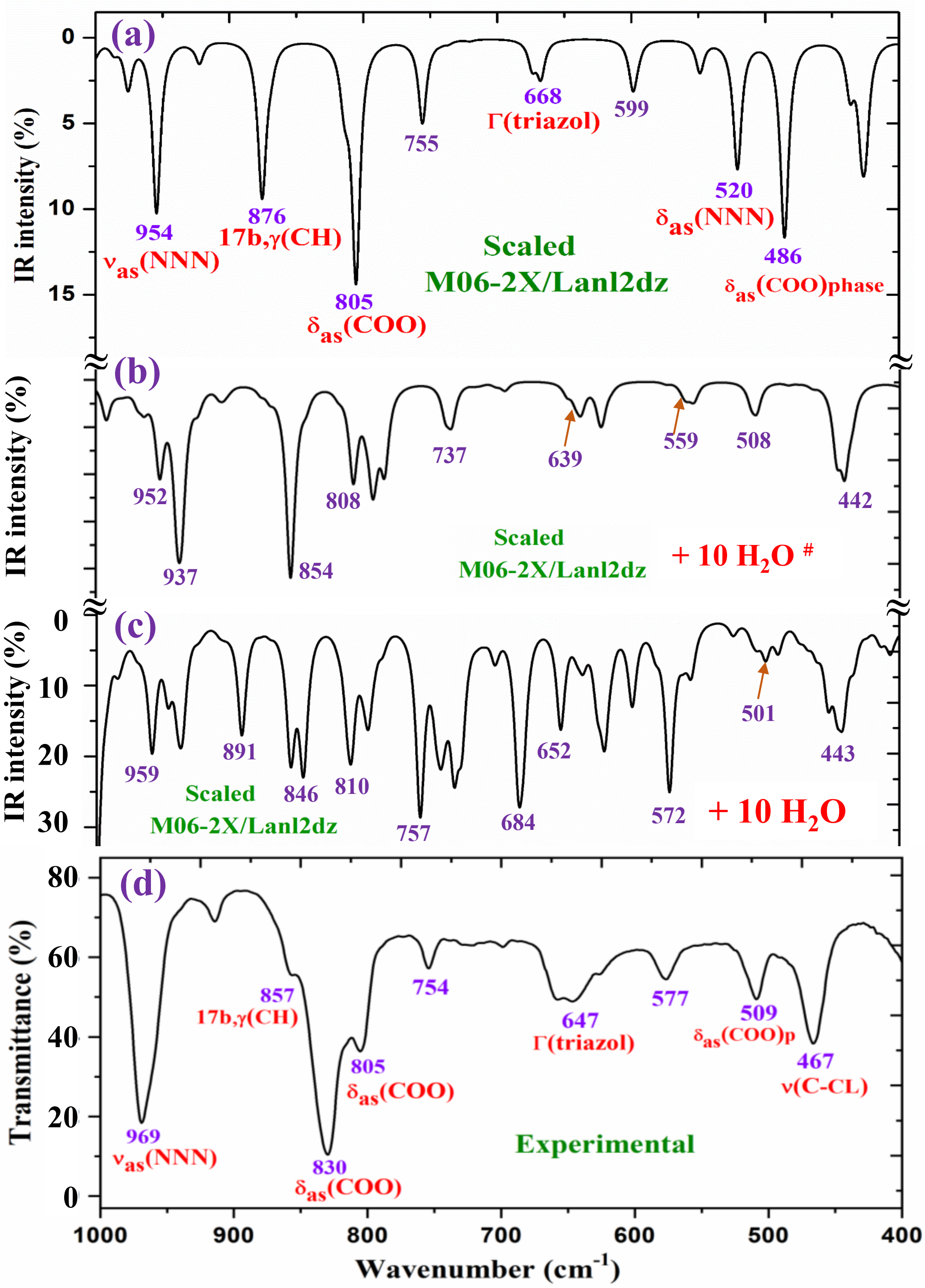

Figure S11. Comparison of the theoretical scaled IR at the M06-2X/Lanl2dz spectra with the experimental ones in the 1000-400 cm^{-1} range. (a) Scaled spectrum in the isolated La(2b′)$_3$ complex. (b) Scaled spectrum in the La(2b′)$_3$ + 10 H_2O cluster but with the water molecule bands subtracted from the spectrum. (c) Scaled spectrum in the La(2b′)$_3$ + 10 H_2O cluster with all bands. (d) Experimental IR spectrum in the solid state sample.

Table S1. Calculated, scaled and experimental wavenumbers (ν, cm^{-1}) in the La$(2b)_3$ complex. Relative infrared intensity (A) in %, relative Raman intensity (S) in %, and Raman depolarization ratios for plane (DP) and unpolarized incident light (DU). For each vibration of the tetramer, the wavenumber with the highest IR intensity is indicated in bold type and that with the highest Raman intensity is indicated in italic type. The relative IR and Raman intensities were only shown for these wavenumbers. DP and DU values were from most intense Raman line. The number of the ring mode corresponds to Wilson's notation [42].

Calculated at **M06-2X/Lanl2dz**						Experimental		
ν	A	S	DP	DU	scaled	IR	Raman	Characterization
						3401.6 br, s		ν(O-H) H_2O bonded
3287, 3287, 3287	0	4	0.13	0.23	3063			2, ν(C3-H) in aryl (100)
3283, 3282, *3281*	0	2	0.11	0.20	3060, 3059			20b, ν(C5-H) in aryl (100)
3266, ***3266***, 3265	0	3	0.57	0.73	3044			7b, ν(C6-H) in aryl (100)
3265, ***3264***, 3264	0	3	0.65	0.79	3043			20a, ν(C2-H) in aryl (100)
3188, 3187, ***3187***	6	3	0.72	0.84	2973	2968.1 s		ν_{as}(C-H) in C16H_2, C17H_2 (100)
3177, ***3177***, 3176	2	4	0.32	0.48	2963			ν_{as}(C-H) in C16H_2, C17H_2 (100)
3168, 3167, ***3167***	0	2	0.57	0.73	2955			ν_{as}(C-H) in C18H_2, C17H_2 pyrrolidine (100)
3144, 3143, ***3143***	2	3	030	0.46	2933			ν_{as}(C-H) in C15H_2 in pyrrolidine (100)
3116, 3116, 3114	4	4	0.03	0.06	2907	2872.2 m		ν_s(C-H) in C18H_2 in pyrrolidine (100)
3107, ***3107***, 3106	2	3	0.19	0.33	2899			ν_s(C-H) in C16H_2 in pyrrolidine (100)
**3103**, 3103, 3102	2	4	0.15	0.27	2895			ν_s(C-H) in C17H_2 in pyrrolidine (100)
3068, **3067**, 3067	4	3	0.10	0.18	2863			ν_s(C-H) in C15H_2 in pyrrolidine (100)
	--	--			--	1895.1 vw		Combination band
1687, 1687, *1686*	0	55	0.39	0.56	1600		1597 vs	8a, ν(C=C) in aryl (89)
1677, ***1677***, 1676	0	0	0.75	0.86	1591			8b, ν(C=C) in aryl (82)
**1666**, *1663*, 1662	100	24	0.06	0.12	**1581**, *1578*	1577.8 br, vs		ν(C_8-N_{14}) (73) + ν_s(N_7CC) (15)
1607, 1596, **1596**	46	100	0.08	0.15	*1527*, **1516**	1500.2 s	1504 s	ν_{as}(COO) (49) + ν(C_9-C_{11})
1550, 1550, 1550	4	9	0.48	0.65	1475			δ(C-H) in-phase in pyrrolidine (92)
1549, *1544*, **1543**	83	5	0.75	0.86	1474, ***1470***	1484.5 vs		19a, ν(CC)(35) + δ(CH) in pyrrolidine (15)
1539, ***1538***, 1538	18	2	0.75	0.86	1465			δ_s(C-H) out-of-phase in pyrrolidine (88)
1527, 1527, 1526	4	0	0.47	0.64	1454			δ_s(C-H) out-of-phase in pyrrolidine (85)
1522, ***1522***, 1521	0	1	0.73	0.85	1449			δ_s(C-H) in pyrrolidine
**1471**, 1471, 1470	2	0	0.64	0.78	1402	1418.0 w		19b,ν(CC,CH) in aryl + ν(C_4N) + ν_s(COO)
**1461**, 1455, *1454*	28	0	0.75	0.86	**1393**, 1388	1398.1 m		ν_{as}(CCOO) + ν_s(NNN) + ν(C-N)
-	--	--	-	-	-	-	-	-
1441, *1440*, **1440**	8	21	0.32	0.48	1375	1372.3 vs	1375 vs	ν_s(COO) + ν_s(NNN) + δ_s(CC,CH)
1426, **1422**, 1422	32	16	0.37	0.53	1361			14, ν(CC) + ν(CO_{12}) + ν(NNC)
1404, **1402**, *1401*	14	2	0.75	0.85	1341, **1340**	1347.5 s		δ_s(C-H) in pyrrolidine (30)
**1392**, *1391*, 1391	0	1	0.45	0.62	1330			γ(C-H) in pyrrolidine (73) + ν_{as}(NNN)
1369, 1368, *1367*	0	0	0.13	0.23	1309, 1307			γ_{as}(CH) in pyrrolidine
1366, 1366, 1366	2	1	0.38	0.55	1306			14, ν(CC) + ν(NN) + δ(C-H) in pyrrolidine
1363, 1363, 1363	2	0	0.38	0.55	1304	1302.0 vs		Γ(pyrrolidine)
1344, 1343, **1341**	0	0	0.75	0.86	1286, **1283**			3, δ(C-H) in aryl
1326, ***1322***, 1321	42	12	0.75	0.86	1270, ***1267***	1285.5 s		ν(NN,CN) + γ_{as}(CC,CH) in pyrrolidine
1291, **1291**, 1290	2	4	0.65	0.79	1238	1246.8 m		ν_{as}(NN,CN)+3,δ(CH) in aryl+δ(pyrrolidine)
1285, ***1283***, 1283	22	2	0.75	0.86	1232, **1230**	1218.0 m		γ_{as}(C-H) out-of-phase in pyrrolidine
1276, ***1275***, 1275	0	0	0.23	0.37	1224			γ_{as}(C-H) in pyrrolidine
1254, **1253**, 1252	4	0	0.73	0.84	1204, **1202**			γ_{as}(C-H) out-of-phase in pyrrolidine (70)
	--	--						
1235, **1233**, *1232*	22	0	0.75	0.86	1186, **1184**	1178.2 m		γ_{as}(C-H) in pyrrolidine
**1219**, 1218, *1216*	0	2	0.29	0.44	**1172**, *1169*		1167 w	9a, δ(C-H) in aryl
1209, **1208**, *1207*	14	1	0.75	0.86	1163, **1162**	1150 sh		γ_{as}(C-H) in pyrrolidine (93)
1205, 1205, 1205	0	0	0.67	0.80	1159			γ_{as}(C-H) in pyrrolidine
1167, 1167, **1166**	0	0	0.23	0.37	1124			γ_{as}(C-H) in pyrrolidine
1149, ***1148***, 1146	0	0	0.55	0.71	1108, **1107**			15, δ(C-H) in aryl
1127, **1127**, *1126*	2	0	0.49	0.66	1088			18a, δ(C-H) in aryl
1123, 1123, 1123	8	5	0.02	0.04	1084	1091.2 vs	1091 m	12, δ(CC,CH) in aryl (96)
1099, 1099, 1099	0	1	0.62	0.76	1062			δ(CC,CH) in pyrrolidine (73)
1041, 1041, 1040	0	0	0.45	0.62	1009			17a, γ(C-H) in aryl
1037, 1037, **1036**	2	1	0.22	0.36	1005	1011.8 m	1013 w	18a, δ(CC, CH) in aryl (98)
1031, 1031, 1030	0	0	0.72	0.84	1000			5, γ(C-H) in aryl (99)
1016, 1016, 1016	0	1	0.75	0.86	986			γ(CC,CH) in pyrrolidine + δ(CN)
**1005**, 1004, *1004*	2	0	0.47	0.64	976			γ_{as}(CC,C-H) in pyrrolidine (86) + δ(CN)

	--	--						
981, 981, 981	10	17	0.07	0.14	954	969.0 vs	970 s	ν_{as}(NNN) + 18a, δ(CC,CH) in aryl
954, 953, 953	0	0	0.65	0.79	929			γ_{as}(C-H) in pyrrolidine (99)
947, **946**, 946	2	1	0.10	0.18	923, **922**	914.3 w		γ_{as}(C-H) in pyrrolidine (97)
900, 900, 900	0	0	0.09	0.17	880			γ_{as}(C-H) in pyrrolidine
**896**, 895, 894	8	0	0.74	0.85	876, 874	857 w-m		17b, γ(C-H) in aryl (100)
888, *888*, **887**	0	1	0.09	0.17	869			γ_{as}(C-H) in pyrrolidine
886, 886, 884	0	0	0.19	0.32	867			10a, γ(C-H) in aryl
830, **828**, 827	4	3	0.04	0.07	*816*, **814**	829.7 vs		γ_{s}(COO) + γ(triazol)
826, **819**, 818	16	3	0.04	0.07	812, **805**	805.4 m		δ_{as}(COO) + γ(C_9-C_{11})
766, **764**, 764	6	0	0.09	0.16	757, **755**	754.3 w		δ_{s}(COO) + 6a, δ(CC) in aryl
750, 750, 750	0	0	0.68	0.81	743			4, γ(CC) in aryl (98)
743, 743, 743	0	0	0.22	0.36	736			6a,δ(CCC) in aryl + γ_{as}(C-H) in pyrrolidine
	--	--						
**726**, *726*, 724	0	0	0.24	0.38	721	698.8 vw		γ_{as}(NNN) (58) + γ_{s}(COO) (22)
675, *675*, **674**	2	0	0.73	0.85	674	654 m		γ_{s}(NNN) + δ_{as}(COO)
**668**, *668*, 667	2	0	0.73	0.84	668	647.1 m		Γ(triazol) (65) + δ_{as}(COO) (18)
648, 647, 647	0	1	0.75	0.86	650			6b, δ(CCC) in aryl
596, **593**, 592	4	2	0.13	0.22	*602*, **599**	576.7 w		δ(NNN) + ν(CCL) + δ(COO) + 6b, δ(CCC)
588, 588, 588	0	0	0.26	0.42	595			γ_{as}(C-H) in pyrrolidine
538, 538, 537	2	0	0.72	0.84	549			16b, γ(CCC) in aryl (35) + γ(NNN)(28)
511, ***507***, 507	8	0	0.74	0.85	524, **520**	466.5 m		δ_{as}(NNN) +ν(CCL) +Γ(CC) aryl +δ_{as}(COO)
472, **469**, 468	12	1	0.34	0.50	489, **486**	508.9 m		δ_{as}(COO) in phase + 6b, δ(CCC) in aryl +La
432, 432, 431	0	0	0.70	0.83	452			10a, γ(CH,CC) in aryl (100)
415, 415, 414	4	0	0.68	0.81	436			
**406**, 403, *402*	10	1	0.74	0.85	**428**, *424*			δ_{as}(COO) in-phase + δ(triazol) + Γ(ring)
366, **365**, 363	2	0	0.70	0.82	392, 389			
326, 325, 325	0	0	0.51	0.67	355			
302, *301*, 299	4	0	0.70	0.82	333, 330			
264, 263, 262	0	0	0.38	0.55	*298*, 296			
251, 250, *250*	0	0	0.67	0.81	286			
241, 238, 234	2	0	0.73	0.84	*277*, 270			
230, *229*, 225	2	0	0.17	0.29	267, 262			
198, *196*, 195	0	0	0.43	0.60	238, 235			
180, 175, *169*	4	0	0.32	0.49	221, 210			
167, 167, 147	0	0	0.71	0.83	*210*, 190			
145, 143, *120*	0	0	0.22	0.36	189, *163*			
113, *106*, 104	0	0	0.72	0.84	160, 151		72 br m	
87, 84, 78	0	0	0.71	0.83	*136*, 127			
75, 74, 70	0	0	0.75	0.86	*125*, 120			
67, 61, *60*	0	0	0.71	0.83	118, *111*			
58, *52*, 51	0	0	0.73	0.84	110, 103			
49, *39*, 39	0	0	0.74	0.85	102, 92			
38, *36*, 35	0	0	0.75	0.86	92, 89			
31, 30, 27	0	0	0.74	0.85	*85*, 81			
8, *7*, 6	0	0	0.75	0.86	64, 62			
5, 4, 3	0	0	0.75	0.86	61, 58			

Table S2. Calculated, scaled and vibrational assignment at the M06-2X/Lanl2mb level, together with the experimental wavenumbers (ν, cm^{-1}) in the La$(2b)_3$ complex. Relative infrared intensity (A) in %, relative Raman intensity (S) in %, and Raman depolarization ratios for plane (DP) and unpolarized incident light (DU). For each vibration of the tetramer, the wavenumber with the highest IR intensity is indicated in bold type and that with the highest Raman intensity is indicated in italic type. The relative IR and Raman intensities were only shown for these wavenumbers. DP and DU values were from most intense Raman line. The number of the ring mode corresponds to Wilson's notation [42].

Calculated at **M06-2X/Lanl2mb**				Experimental		
ν	A	S	scaled	IR	Raman	Characterization
		--	--	3401.6 br, s		ν(O-H) H_2O bonded
3556, 3556, 3556	3	11	2958			2, ν(C_3-H) in aryl (100)
3555, 3555, 3555	1	11	2957			7b, ν(C_5-H) in aryl (100)
3553, 3553, 3553	26	3	2956	2968.1 s		20b, ν(C_6-H) in aryl (100)
3549, 3549, 3548	0	4	2953			20a, ν(C_2-H) in aryl (100)
3541, 3541, 3540	1	5	2948			ν_{as}(C-H) in $C16H_2$, $C17H_2$ (100)

3536, 3536, 3536	4	1	2944			ν_{as}(C-H) in $C_{16}H_2$, $C_{17}H_2$ (100)
3499, 3498, 3489	0	3	2919			ν_{as}(C-H) in $C_{18}H_2$, $C_{17}H_2$ pyrrolidine (100)
3488, 3468, ***3453***	14	16	2911, ***2892***	2872.2 m		ν_{as}(C-H) in $C_{15}H_2$ pyrrolidine (100)
3439, 3439, 3439	0	8	2878			ν_s(C-H) in $C_{18}H_2$ pyrrolidine (100)
3437, 3437, *3435*	0	3	2876			ν_s(C-H) in $C_{16}H_2$ pyrrolidine (100)
3387, **3387**, 3383	1	4	2842			ν_s(C-H) in $C_{17}H_2$ pyrrolidine (100)
3380, 3367, ***3350***	16	14	2837			ν_s(C-H) in $C_{15}H_2$ pyrrolidine (100)
	--	--	--	1895.1 vw		Combination band
1773, 1773, 1773	0	1	1621			8b, ν(C=C) in aryl (91)
1769, 1769, 1769	9	97	1617		1597 vs	8a, ν(C=C) in aryl (93)
1716, 1701, 1700	83	22	***1574***, 1560	1577.8 br, vs		$\nu(C_8-N_{14}) + \nu_s(N_7CC)$
1697, 1692, **1689**	32	14	*1558*, **1552**			δ(C-H) in pyrrolidine + $\nu(C_8-N_{14})$
1686, 1681, 1680	18	9	***1549***, 1544			δ(C-H) in pyrrolidine + $\nu(C_8-N_{14})$
1679, **1671**, *1670*	15	2	1543, ***1536***			δ_s(C-H) in pyrrolidine
1669, 1668, 1668	11	4	1535			δ_s(C-H) out-of-phase in pyrrolidine (85)
1640, 1640, 1639	55	100	1511	1500.2 s	1504 s	19a, ν(CC,CH) + $\nu(C_4-N_4)$ +$\nu(C_9-C_{11})$
1616, *1602*, **1595**	100	80	1491, *1477*, **1472**	1484.5 vs		ν_{as}(COO)+ν_s(CCN)triazol+$\nu(C_9-C_{11})$+19a,ν(CC)
1576, 1562, 1555	17	14	***1458***, 1441			ν_{as}(COO) + ν_s(CCN) in triazol
1550, *1548*, **1547**	6	9	1436. ***1433***			19b, ν(CC,CH) + ν_s(NNN) + ν_{as}(COO)
1518, **1510**, 1509	14	2	*1409*. **1401**	1418.0 w		δ_s(C-H) in pyrrolidine + ν_s(NNN) + ν_{as}(COO)
1501, ***1500***, 1498	7	16	***1395***, 1392	1398.1 m		δ_s(C-H) in pyrrolidine + ν_s(NNN)
1494, **1491**, *1486*	4	25	1389, **1386**, *1382*			δ_s(C-H) in pyrrolidine + ν_s(NNN)
1484, ***1477***, 1475	19	33	1380, ***1375***, 1373		1375 vs	ν_s(NNN) + ν_s(COO) + γ_s(C-H) in pyrrolidine
1474, **1469**, 1468	71	18	*1372*, **1368**	1372.3 vs		ν_s(COO) +ν_s(NNN) + δ_s(CC,CH) +19a, ν(CC)
1433, 1425, 1424	15	1	***1337***, 1329	1347.5 s		Γ(C-H) in pyrrolidine
1399, **1394**, *1390*	94	16	1309, **1304**, *1301*	1302.0 vs		ν_{as}(NNN)(36) + δ(C-H) in pyrrolidine (30)
	--	--				
1389, 1387, ***1386***	9	5	1300, ***1297***			3, δ(C-H) + ν(triazol) + δ(C-H) in pyrrolidine
1367, **1365**, 1362	50	16	*1281*, **1279**, 1276	1285.5 s		δ_{as}(C-H) in pyrrolidine + ν(triazol)
1351, 1348, 1347	48	7	***1268***, 1265			ν(triazol) + 3, δ(CH)+ δ(C-H) in pyrrolidine
1343, 1343, 1343	2	1	1261			ν(pyrrolidine)
1339, ***1337***, 1336	82	8	1258, ***1256***	1246.8 m		ν(triazol)+ 3, δ(C-H) +δ(C-H) in pyrrolidine
1327, **1318**, 1316	11	3	*1247*, **1239**, 1237			δ(CC,CH) in pyrrolidine
1312, 1311, 1311	17	0	1234	1218.0 m		14,ν(CC) in aryl +ν_{as}(NN)+δ(C-H) pyrrolidine
1280, 1278, ***1274***	16	2	1207, 1205, ***1202***			ν_s(COO) + δ(triazole) + δ_{as}(C-H) pyrrolidine
**1269**, *1259*, 1256	22	1	**1198**, *1189*, 1186	1178.2 m		ν(triazole) + γ_{as}(C-H) in pyrrolidine+ν_s(COO)
	--	--				
1230, 1229, 1229	1	5	1164		1167 w	9a, δ(C-H) in aryl
1225, 1219, 1216	2	1	***1160***, 1154, 1151	1150 sh		δ_{as}(C-H) in pyrrolidine
1186, 1186, 1186	35	2	1126			18a, δ(C-H) in aryl
1170, 1170, 1170	1	0	1112			15, δ(C-H) in aryl
1165, 1165, 1163	1	1	***1108***, 1106			δ_{as}(C-H) in pyrrolidine
1155, 1154, **1153**	3	1	1099, **1097**	1091.2 vs	1091 m	ν_s(NNN) + 18a, δ(C-H) in aryl
1074, 1073, 1073	1	3	1029			δ_{as}(C-H) in pyrrolidine
1071, 1070, 1068	1	3	***1026***, 1023			18a, δ(C-H) in aryl
1068, 1067, 1067	0	0	1023			12, δ(CC, CH) in aryl + δ(CH) + ν_s(triazol)
**1054**, *1050*, 1050	3	1	**1011**, *1007*	1011.8 m	1013 w	ν_{as}(triazol)+δ(CC,CH) pyrrolidine +δ_s(COO)
1047, 1047, 1046	0	1	1005			17a, γ(C-H) in aryl
1035, ***1016***, 1016	0	1	994, ***977***			γ(C-H) in pyrrolidine
1006, 1004, ***1003***	1	3	969, ***966***			γ(C-H) in pyrrolidine
	--	--				
974, 973, 973	36	24	941	969.0 vs	970 s	ν_{as}(NNN, CC) in triazol +18a, δ(C-H) in aryl
953, 941, 941	3	5	***922***, 912	914.3 w		γ_{as}(CC,C-H) in pyrrolidine
917, 904, *889*	1	1	***890***, 882			γ_{as}(CC,C-H) in pyrrolidine
907, 906, 906	0	1	881, ***879***, 877			10a, γ(C-H) in aryl
902, 901, **901**	9	0	**876**, *874*	857 w-m		17b, γ(C-H) in aryl
846, **842**, 840	36	3	*827*, **824**, 822	829.7 vs		δ_{as}(COO) + γ_{as}(C-H) pyrrolidine +ν(triazol)
799, ***792***, 789	26	1	785, ***779***, 777	805.4 m		δ_{as}(COO) + γ_{as}(C-H) pyrrolidine +ν(triazol)
777, 745, **773**	1	1	*766*, **763**			γ_{as}(C-H) in pyrrolidine + 6a, δ(CCC) in aryl
766, 764, **761**	7	2	*756*, 754, **752**	754.3 w		$\gamma_s(C_9-C_{11}-C_8)$ + γ_s(COO)
753, 753, 753	0	0	744			4, γ(CCC) in aryl
**687**, *685*, 679	3	1	**684**, *682*, 676	698.8 vw		γ_s(COO) + $\gamma(C_8-C_9)$
658, 658, 658	0	1	658	654 m		6a, δ(CC) in aryl
	--	--				
**638**, *636*, 633	2	0	**640**, *638*, 635	647.1 m		Γ(triazol) + γ_{as}(COO) + γ(C-H) pyrrolidine
630, 580, ***576***	2	2	633, 583, ***579***			Γ(triazol) + γ_{as}(COO) + γ(C-H) pyrrolidine

621, 618,-***583***	5	2	624, 621 ***590***	576.7 w		γ(C-H) in pyrrolidine
559, ***559***, 556	1	0	***568***, 565			γ_s(NNN)
521, 521, 521	0	0	533			16b, γ(CCC) in aryl
**504**, *500*, 489	32	3	**518**, *514*, 503	508.9 m		δ_{as}(COO)phase+δ_{as}(triazol)+ν(CCL)+6b,δ(CCC)
462, **460**, 457	13	1	*479*, **477**, 474	466.5 m		ν(CCL) + δ_{as}(COO) + δ(NNN) + 6b,δ(CCC)
436, 435, 435	0	0	455			16a, γ(CCC) in aryl
421, 411, 399	20	1	***441***, 431, 419			δ(COO) + δ(triazol) + Γ(CC) in aryl
388, **385**, 382	2	1	*411*, **408**, 405			δ(COO) + δ(triazol) + Γ(CC) in aryl
364, **362**, *354*	1	1	388			γ(COO) + γ(triazol) + γ(CC) in aryl
326, *323*, **318**	1	0	353			τ(aryl) + γ(CCN)
	--	--				
316, 292, **283**	7	1				
277, *272*, **267**	2	0				La
**260**, 251, 243	1	0				
237, **234**, 226	0	0				
225, 219, ***213***	1	0				
**198**, *187*, 178	2	0				
167, 161, **159**	1	1				
155, 153, 145	1	0				
138, 131, ***129***	1	0				
125, ***121***, 112	1	0			72 br m	
100, 95, 84	0	0				
80, 74, 70	0	0				
66, 62, 59	0	0				
56, 50, *46*	0	0				
41, 41, *37*	0	0				
35, 33, *32*	0	0				
31, *27*, 23	0	0				
15, 11, 9	0	0				
7, 4, 4	0	0				

Table S3. Calculated, scaled and vibrational assignment at the B3LYP/Cep-4g level, together the experimental wavenumbers (ν, cm^{-1}) in the La(2b)$_3$ complex. Relative infrared intensity (A) in %, relative Raman intensity (S) in %, and Raman depolarization ratios for plane (DP) and unpolarized incident light (DU). For each vibration of the tetramer, the wavenumber with the highest IR intensity is indicated in bold type and that with the highest Raman intensity is indicated in italic type. The relative IR and Raman intensities were only shown for these wavenumbers. DP and DU values were from most intense Raman line. The number of the ring mode corresponds to Wilson's notation [42].

Calculated at **B3LYP/Cep-4g**				Experimental		
ν	A	S	scaled	IR	Raman	Characterization
	--	--		3401.6 br,s		ν(O-H) H_2O bonded
3324, 3323, 3323	5	2	2906			2, ν(C_3-H) in aryl (100)
3314, 3313, 3313	1	2	2899			7b, ν(C_5-H) in aryl (100)
3302, 3302, 3302	8	5	2891			20b, ν(C_6-H) in aryl (100)
3296, 3296, 3296	52	1	2886	2968.1 s		20a, ν(C_2-H) in aryl (100)
3285, ***3283***, 3282	10	1	2879, **2877**			ν_{as}(C-H) in $C_{16}H_2$, $C_{17}H_2$ (100)
3282, 3279, **3278**	3	2	2876, **2874**			ν_{as}(C-H) in $C_{16}H_2$, $C_{17}H_2$ (100)
**3277**, 3276, *3271*	3	1	**2873**, **2869**			ν_{as}(C-H) in $C_{18}H_2$, $C_{17}H_2$ pyrrolidine (100)
**3275**, 3275, **3273**	13	1	2871, **2870**	2872.2 m		13, ν(C-H) in aryl
3211, 3210, 3210	8	5	2826			ν_s(C-H) in $C_{16}H_2$. $C_{17}H_2$ pyrrolidine (100)
3204, 3203, ***3202***	2	2	2821, **2820**			ν_s(C-H) in $C_{16}H_2$ pyrrolidine (100)
**3181**, 3178, *3171*	1	4	**2804**, 2797			ν_s(C-H) in $C_{18}H_2$ pyrrolidine (100)
3169, *3169*, **3167**	4	1	2795, **2793**			ν_s(C-H) in $C_{15}H_2$ pyrrolidine (100)
	--	--	--	1895.1 vw		Combination band
1596, 1595, 1595	35	1	1512	1577.8br,vs		ν(C_8-N_{14}) +8a, ν(C=C) in aryl
1595, *1593*, **1593**	13	100	1511, ***1509***	1500.2 s	1597 vs	8a, ν(C=C) in aryl + ν(C_8-N_{14})
1593, **1591**, 1586	35	58	*1509*, **1507**, 1503	1484.5 vs	1504 s	8b, ν(C=C) in aryl + ν(C_8-N_{14}) + ν_s(N7CC)
1505, **1495**, 1493	57	58	*1429*, **1420**, 1418	1418.0 w		ν_s(COO) (49) + ν(C_9-C_{11})
1462, 1458, 1457	2	2	***1389***, 1386	1398.1 m		δ(C-H) in-phase in pyrrolidine (92)
1443, 1440, **1438**	19	18	*1372*, 1370, **1368**	1372.3 vs	1375 vs	δ(C-H) in pyrrolidine
1437, *1435*, **1435**	100	16	1366, ***1364***	1347.5 s		ν(C_4-N_4), 19a,ν(CC) + δ_s(C-H) in pyrrolidine
1428, 1427, 1427	7	1	1358			19a, ν(CC,CH) in aryl
1421, 1420, 1420	1	0	1351			ν_{as}(COO) + ν_s(CCN) in triazol +19a, ν(CC)
1373, 1373, 1373	1	1	1307			ν(C_4-N) + ν_s(COO)

1370, *1363*, **1356**	6	0	1304, *1298*, **1292**	1302.0 vs		ν_{as}(CCOO) + ν_s(NNN) + ν(C-N)
1336, **1334**, 1334	2	1	1272, **1270**	1285.5 s		19b, ν(CC,CH)+ ν(triazol)+δ_s(C-H) pyrrolidine
1328, **1327**, *1326*	5	0	1265, ***1264***			δ(CH)pyrrolidine +ν(N_7-C)+19b, ν(CC,CH)
1307, 1305, 1304	3	12	***1245***, 1243	1246.8 m		ν(CNN) + 3,δ(CH) + δ(CH) in pyrrolidine
1304, 1302, **1301**	3	9	*1242*, **1240**			δ(C-H) in pyrrolidine + δ(C_8-N_{14})
1295, 1295, 1294	2	6	1234	1218.0 m		δ_s(CC,CH) in pyrrolidine
1284, 1282, ***1282***	2	1	1223, ***1221***			ν(NNC)+ δ_s(CC,CH) pyrrolidine +14, ν(CC)
1271, **1270**, 1267	18	1	***1211***, 1208	1178.2 m		δ(CN) + γ(C-H) in pyrrolidine
	--	--				
1234, **1232**, 1231	10	0	*1176*, **1174**			δ(C-H) in pyrrolidine + ν_{as}(NNN)
1220, **1218**, 1216	35	3	*1163*, **1161**, 1159	1150 sh	1167 w	ν_s(triazol) + δ(C-H) in pyrrolidine
1187, **1186**, *1185*	1	1	1131, ***1129***			14, ν(CC) + ν(NN) + δ(C-H) in pyrrolidine
1182, *1180*, **1179**	23	0	1127, ***1125***	1091.2 vs		ν_s(NNC) + 3, δ(C-H) in aryl
1173, **1173**, 1171	2	1	***1118***, 1116			γ_{as}(C-H) in pyrrolidine (98)
1154, 1153, **1152**	1	0	1100, **1098**			ν(NN,CN)(68) + γ_{as}(CC,CH) in pyrrolidine
1148, 1147, ***1146***	10	0	1094, ***1092***		1091 m	3, δ(C-H) in aryl + δ_{as}(CNN)
1119, **1118**, 1114	1	0	***1066***, 1062			γ_{as}(C-H) out-of-phase in pyrrolidine (96)
1084, *1079*, **1078**	2	1	1032, ***1027***			γ_{as}(C-H) in pyrrolidine + ν_s(CN) + ν_s(COO)
1062, 1062, 1062	0	4	1011		1013 w	9a, δ(C-H) in aryl
1061, **1060**, 1058	1	1	***1010***, 1008	1011.8 m		γ_{as}(C-H) in pyrrolidine
1032, 1032, 1031	1	0	982			γ_{as}(C-H) in pyrrolidine
1026, 1026, 1026	3	1	976	969.0 vs	970 s	18a, δ(C-H) in aryl
1001, 1001, 1000	1	0	952			18b, δ(C-H) in aryl
	--	--				
983, 982, **981**	5	1	*934*, **932**	914.3 w		ν_s(NNN) + 18a, δ(C-H) in aryl
945, 944, ***943***	1	0	897, ***895***			15, δ(C-H) in aryl
913, 913, 913	2	0	866	857 w-m		γ(C-H) in pyrrolidine + ν(CN)
902, 901, 901	2	1	855	829.7 vs		18a, δ(CH) + ν(CN) + γ(C-H) in pyrrolidine
**882**, *879*, 878	0	0	**835**, *832*			δ(CC,CH) in pyrrolidine (73)
873, *872*, **871**	1	1	*826*, **824**	805.4 m		17a, γ(C-H) in aryl
820, ***819***, 817	3	3	***773***, 771			γ(C-H) in pyrrolidine
815, 815, 815	0	0	768			5, γ(C-H) in aryl (95)
811, 810, 810	5	11	764	754.3 w		ν_s(NNN, CC) in triazol +18a, δ(CC, C-H)
806, 806, 806	0	0	759			γ_{as}(CC,C-H) in pyrrolidine + δ(CN)
804, 803, 803	1	1	757			ν_s(NNN) + 18a, δ(CC,CH) in aryl
744, 744, 744	0	0	697	698.8 vw		γ_{as}(C-H) in pyrrolidine (93)
711, 710, 710	20	0	664	654 m		17b, γ(C-H) in aryl
703, **699**, 698	5	5	*656*, **653**	647.1 m		δ_{as}(COO) +γ_{as}(C-H) pyrrolidine + ν(triazol)
653, **650**, 649	2	3	*605*, **602**			δ_{as}(COO) + ν(triazol) + γ_{as}(C-H) pyrrolidine
649, *648*, **645**	15	1	*601*, **597**	576.7 w		δ_{as}(COO) +ν_{as}(triazol)+ γ_{as}(C-H) pyrrolidine
**586**, *584*, 583	2	0	**537**, *535*			10a, γ(C-H) in aryl
544, 543, **542**	1	0	*494*, **492**	508.9 m		δ(CC) + δ_s(COO) + δ(triazol)
533, 533, 533	1	0	483			γ_s(C-H) in pyrrolidine
533, 533, 532	1	0	483			γ_s(C-H) in pyrrolidine + 6a, δ(CC) in aryl
522, 522, 522	0	0	471			4, γ(CCC) in aryl
519, **515**, 511	6	2	*468*, **464**	466.5 m		γ(COO) in phase + 6b, δ(CCC) + δ(CN)
498, 496, ***495***	2	0	446, ***443***			γ_{as}(NNN) + γ_s(COO)
452, 452, 451	0	0	399			γ_s(NNN) + δ_{as}(COO)
**443**, 440, *433*	25	1	389			δ_{as}(COO) + La + γ(triazol)
**403**, *401*, 400	2	0	348			γ(triazol) + ν(CCL) + 6b, δ(CCC) in aryl
	--	--				
390, 390, 389	1	0	334			δ(NNN) + ν(CCL) + 6b, δ(CC) in aryl
366, 358, **354**	4	0	309			γ_{as}(C-H) in pyrrolidine
339, 338, ***334***	2	1	281			δ_{as}(COO) phase +δ(NNN) +ν(CCL) + Γ(ring)
326, 326, 326	0	0	267			δ(NNN) + δ(CCL) + δ(CC) aryl + δ(COO)
303, ***302***, 301	1	0	243			δ(COO) structure + δ(aryl) + δ(NNN) + La
265, **264**, *260*	0	0	202			10a, γ(CC) in aryl
256, **253**, *251*	1	0	193			
233, 231, 226	2	0	168		72 br m	La
**220**, 217,*216*	1	0	154			
204, 202, 200	0	0	137			
197, *194*, **191**	1	0	130			
**168**, 167, *163*	1	0	99			
150, 149, 149	0	0	80			
**142**, *140*, 134	0	0	71			
133, 130, *127*	0	0	61			

110, **106**, 103	0	0	36			
87, ***84***, 75	0	0	12			
71, **63**, *60*	0	0				
53, 50, *46*	0	0				
45, 44, **42**	0	0				
**40**, 36, *32*	0	0				
**31**, 30, **27**	0	0				
26, 26, 23	0	0				
**17**, 11, *9*	0	0				
6, 4, *4*	0	0				
